# Environmental Factors Affecting Diversity, Structure, and Temporal Variation of Airborne Fungal Communities in a Research and Teaching Building of Tianjin University, China

**DOI:** 10.3390/jof8050431

**Published:** 2022-04-22

**Authors:** Yixuan Lu, Xiao Wang, Lucineidy C. S. de S. Almeida, Lorenzo Pecoraro

**Affiliations:** School of Pharmaceutical Science and Technology, Tianjin University, 92 Weijin Road, Tianjin 300072, China; luyixuann2022@163.com (Y.L.); wang_xiao1996@163.com (X.W.); lu.kaili551@gmail.com (L.C.S.d.S.A.)

**Keywords:** airborne fungi, fungal diversity and community structure, ITS sequencing, morphological analysis, environmental factors, indoor and outdoor environments

## Abstract

Airborne fungi are widely distributed in the environment and may have adverse effects on human health. A 12-month survey on the diversity and concentration of culturable airborne fungi was carried out in a research and teaching building of Tianjin University. Indoor and outdoor environments were analyzed using an HAS-100B air sampler. A total of 667 fungal strains, belonging to 160 species and 73 genera were isolated and identified based on morphological and molecular analysis. The most abundant fungal genera were *Alternaria* (38.57%), *Cladosporium* (21.49%), and *Aspergillus* (5.34%), while the most frequently appearing species was *A. alternata* (21%), followed by *A. tenuissima* (12.4%), and *C. cladosporioides* (9.3%). The concentration of fungi in different environments ranged from 0 to 150 CFU/m^3^ and was significantly higher outdoor than indoor. Temperature and sampling month were significant factors influencing the whole building fungal community, while relative humidity and wind speed were highly correlated with fungal composition outdoor. Variations in the relative abundance of major airborne fungal taxa at different heights above-ground could lead to different community structures at different floors. Our results may provide valuable information for air quality monitoring and microbial pollution control in university building environments.

## 1. Introduction

Among the variety of biological particles present in the atmosphere, fungal spores represent a major component of both indoor and outdoor air environments [1], which is associated with air pollution and human health problems [2,3]. Excessive inhalation of harmful airborne fungi can cause respiratory symptoms, pulmonary infections, and result in compromised immune system [4,5,6,7]. Until now, more than 80 fungal genera have been associated with respiratory allergies [2,8], and a total of approximately 150 allergenic fungal species have been identified, while at least 3% to 10% of the global population is affected by fungal sensitization [9]. The most common allergenic fungal genera are *Alternaria, Cladosporium, Aspergillus,* and *Fusarium* [2,6]. For example, several clinical studies and epidemiological research have shown a highly variable prevalence of human Immunoglobulin E (IgE) reactivity to *Alternaria* [10,11,12,13]. The European Community Respiratory Health Survey found that more than 4% of the adult population (*n* = 11,355) in 13 different countries was sensitized to *Alternaria* [14]. A study performed in Australia showed that asthma, triggered by exacerbations of airway responsiveness, wheezing, and bronchodilation, was associated with elevated concentrations of *Alternaria* in the air [15]. Fungal species belonging to the genus *Cladosporium* were found to be the third most common cause of sensitization, after house dust mite and grass pollen, by using skin-prick tested with a battery of allergens among 1218 children born on the Isle of Wight [5]. According to a study conducted in Poland, the highest intensities of *Cladosporium*-associated allergic symptoms were recorded in summer and autumn [16]. Antigen-specific IgE and histamine release test performed in Spain on a total of 2200 patients diagnosed with rhinosinusitis and/or bronchial asthma, showed that 26% of tested persons presented sensitization to *Cladosporium* [17]. Some researchers have shown that various airborne fungi, such as *Aspergillus*, *Fusarium*, and *Mucorales* taxa, can cause hospital infections [18,19,20]. *Aspergillus* fungi are pathogenic to several animal and plant species, including humans and domestic animals. For instance, *A. flavus* is an opportunistic fungal pathogen that causes invasive aspergillosis, a usually fatal infection in humans, by colonizing lungs or respiratory tracts. Patients infected with *A. flavus* often show reduced or compromised immune systems [21]. *Fusarium* fungal species are emerging human pathogens frequently isolated from cornea and less commonly from nail, skin and blood, especially among immunocompromised haematology patients [22]. In a case study, skin testing with fungal extracts, combined with high resolution computerized tomography, was used to realize an enzyme linked immunosorbent assay (ELISA), which helped to detect serum IgE and IgG antibodies to *F. vasinfectum* among 12-year-old children affected by allergic bronchopulmonary aspergillosis (ABPA) [23]. In 2009, epidemiologic studies reported that 8.2% of adults and children (24.6 million persons) in the United States were afflicted by asthma associated with sensitivity to airborne fungi, particularly to *A. alternata* and *C. herbarum* [24,25]. Sensitization to fungi is a significant factor in patients with allergic respiratory tract problems, playing a major role in the development, persistence, and severity of lower airway diseases [26]. Some studies have demonstrated that people exposure to airborne fungi in indoor environment can aggravate allergic diseases [24,27,28,29,30], while high concentration of fungal particles in the air has been linked to human health adverse effects [27,30]. It has been shown that people spend more than 85% of their life time in indoor environments [27], where airborne bacteria and fungi are generally introduced from the outdoor environment via natural ventilation system, such as windows and doors [27,31], mechanical ventilation system such as heating, ventilating and air conditioning (HVAC) systems [31], and human occupancy [32]. Previous studies have found that fungal concentrations outdoor were higher than those in indoor environments [33,34,35,36]. Concerning outdoor environments, many epidemiological studies have shown that airborne fungi can cause serious respiratory problems [37,38,39,40,41,42,43,44,45,46,47,48,49]. For indoor environments, airborne fungi-induced health issues have attracted much attention, especially in public buildings [40]. Many researches have characterized fungal bioaerosols and assessed exposure risks in various communal buildings, including hospitals [41,42,43], libraries [44,45], and offices [46]. A better understanding of the fungal bioaerosol community dynamics in human dwellings can be crucial to evaluate potential health hazards [9,47].

The diversity and concentration of fungal spores in the atmosphere depends on various factors including sampling locations, seasons, and flow of people [48,49]. For instance, Kim et al., (2009) investigated the distribution patterns of airborne bacteria and fungi in feed stuff-manufacturing areas, showing a correlation between relative humidity and airborne microorganism’s bioactivity [50]. Dong et al., (2016) found that the diversity of airborne fungi associated with particulate matters (PMs) in Beijing varied significantly among PM sizes and air quality levels [51]. Morin et al., (2020) analyzed the concentrations of indoor airborne fungi throughout a whole year in a hematology ward in Japan, showing that seasonal changes affected the presence and dispersion of airborne fungi, with the highest number of fungal colonies recorded in October [52]. Burge et al., (1995) examined seasonal variation of indoor allergen concentrations in Boston and found that the presence of airborne fungi was closely associated with relative humidity, being levels above 70%, optimal for fungal growth [53].

The assessment of microbial air quality in public buildings characterized by complex structure and by the presence of various human activities, such as schools and universities, is very important to define potential health risks related to fungal pollution [40]. Wu et al., (2020) investigated the concentration of airborne fungi in the University of Xi’an, China, which was significantly affected by seasonal variation in all analyzed rooms, except for the reading room [44]. Kalwasińska et al., (2012) recorded the number of mould fungi in both indoor and outdoor environment of Toruń University Library, in Poland, and revealed that the concentrations of fungal aerosol particles ranged between 10 and 10^3^ CFU/m^3^ [25]. The aim of our study was to investigate the diversity and concentration of airborne fungi in different indoor and outdoor environments in the teaching and research building 24 of Tianjin University, Tianjin, China. We compared the indoor and outdoor airborne fungal concentrations and analyzed the relevant environmental parameters affecting the diversity of the analyzed fungal community. Extending our knowledge of biological diversity of airborne fungi in university buildings may be of pivotal importance to provide guidelines for air quality control and management in our living environments.

## 2. Materials and Methods

### 2.1. Study Sites and Sampling

**Figure 1:**
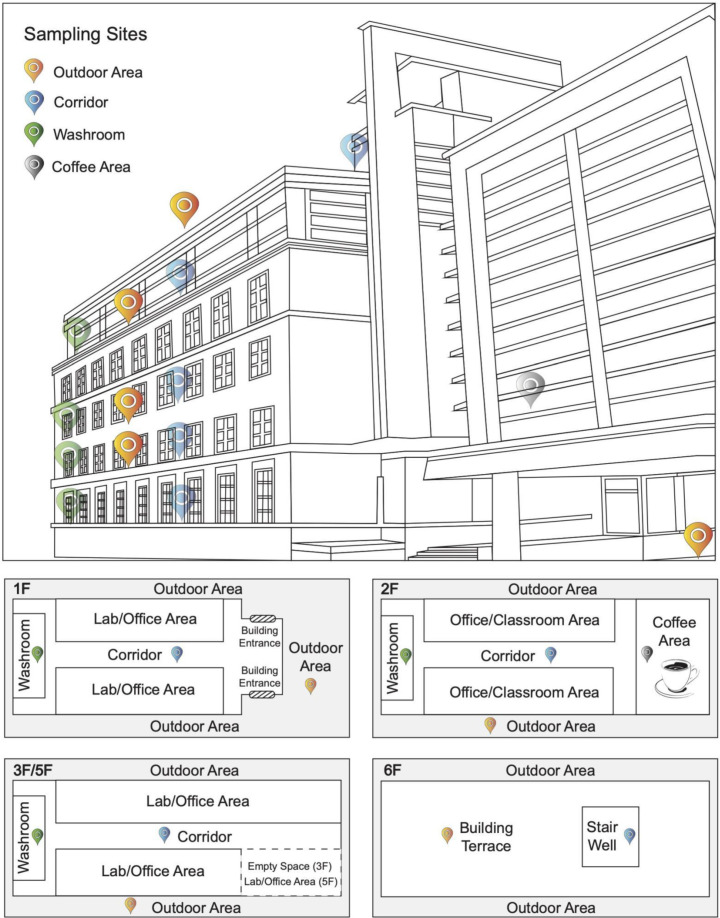
Schematic diagrams of Tianjin University building 24 (front view) and sampling floors (vertical view). Half of the building and floors were displayed, since the layout of the building and each floor are practically symmetrical. The fifteen sampling sites are marked in the diagrams.

This study was conducted in Tianjin city, located in the North-East of China. The sampling sites were located in the building number 24 of Tianjin University, on Weijin Road, Nankai District (39°6′32″ N, 117°10′11″ E). The building 24 is a comprehensive teaching and research building, with classrooms and study rooms for students, offices, equipment rooms, research laboratories, and a coffee area. Fifteen sampling sites were selected in the study building to represent different environments, characterized by different functions. These included:(i) corridors, which have a central position at each floor, and provide access to laboratories and offices from both sides, with the exception of the sixth floor, which is constituted by a rooftop where the corridor environment is simply reduced to a stair well to access the building terrace, (ii) washrooms, located at the two extremities of the building in a symmetrical position at each floor, (iii) the coffee area located at second floor, in the center of the building, and (iv) outdoor environments, accessed through the windows at floors 1, 2, 3, and 5, and through the terrace at floor 6 (Figure 1).

Sampling of airborne fungi at different locations was conducted for a whole year, from May 2020 to April 2021, once a month, when the weather parameters were stable, approximately between 9:00 and 12:00 in the morning. An air sampler HAS-100B (Beijing, China) was used to collect the culturable airborne fungi from the selected environments, using sterile plates of 9 cm diameters containing Malt Extract Agar (MEA) added with ampicillin (100 mg/L) to inhibit the proliferation of bacteria. At each sampling location, the air sampler, fixed on a support at a height of 1.5 m above ground, was operated for 1 min with an air flow of 100 L/min and rotating dish speed of 0–4 rpm. Before each sampling, the sampler was swabbed using 70% ethanol to prevent cross-contamination. Environmental parameters, including Temperature (T) and Relative Humidity (RH) were measured at each sampling site, using a TES 1364 Humidity-Temperature Meter (Taiwan, China). Data on wind speed and air quality index were retrieved from https://weather.com (accessed on 21 January 2022). After collecting the air samples, the exposed agar plates were incubated at 25 °C, in the darkness, and examined for fungal colony growth every 24 h for 1 week. For pure cultures, colonies appearing on the primary MEA plates were counted, carefully picked up and isolated into new plates. Morphology of mycelial growth was observed every day. The data related to the number of fungal colonies detected at each studied site and month were transformed to fungal concentrations and expressed as colony forming units per cubic meter of air (CFU/m^3^) [2], which was calculated as:

(1) $$N=\frac{Cn*1000}{t*V}$$

where *Cn* = Number of fungal colonies, *t* = sampling time, *V* = velocity of air flow.

All isolated fungal strains were deposited in the LP Culture Collection (personal culture collection held in the laboratory of Prof. Lorenzo Pecoraro) at the School of Pharmaceutical Science and Technology, Tianjin University, Tianjin, China.

### 2.2. Fungal Identification

All the isolated strains were identified based on molecular and morphological analyses. For the molecular identification, DNA extraction was conducted using the cetyltrimethylammonium bromide method [54]. After the DNA extraction, fungal DNA was amplified by polymerase chain reaction (PCR) using the following set of primers: ITS1 (5′-TCCGTAGGTGAACCTGCGG-3′) and ITS4 (5′TCCTCCGCTTATTGATATGC-3′) [55]. The reaction mixture for PCR (50 µL), consisted of 25.0 µL of 2× Rapid Taq Master Mix (Vazyme Biotech Co. Ltd., Nanjing), 2 µL of forward primer (10 µM), 2 µL of reverse primer (10 µM), 2.0 µL (20 ng DNA) of template, and 19 µL of double distilled sterilized water. The PCR amplifications were performed by set-up program: initial denaturation at 98 °C for 3 min, 30–35 cycles of 98 °C for 10 s, annealing at 55 °C for 15 s, extension at 72 °C for 15 s, and final extension at 72 °C for 2 min. Gel electrophoresis using electrophoresis tank (LiuYi, Beijing) on a 1% agarose gel was performed to detect the PCR products. Sequencing of the PCR products was performed at Tsingke Biological Technology Company (Beijing, China). Basic Local Alignment Search Tool (BLAST) program of the National Center for Biotechnology Information, USA (http://www.ncbi.nlm.nih.gov/Blast.cgi, accessed on 12 February 2022) was used to analyze each DNA sequence in order to identify the closest match in the NCBI database. DNA sequences were deposited in GenBank (Accession No. ON208139—ON208805). Fungal morphology was characterized based on macroscopic and microscopic observations. Microscopy was done using a Nikon ECLIPSE Ci-L microscope (Tokyo, Japan) to examine fungal morphological characters, including hyphae, conidiophores, conidia, poroconidia, arthroconidia, etc.

### 2.3. Statistical Analysis

The composition of airborne fungal communities identified from all sampling sites were illustrated by Krona Charts, which were prepared using Krona Tools (https://github.com/marbl/Krona/wiki/KronaTools, accessed on 11 March 2022) [56]. Fungal diversity was evaluated using Sobs, Shannon and Chao1 indexes. Between-group Venn diagrams [57] were plotted using R to identify unique and common fungal genera. Principal Co-ordinates Analysis (PCoA) and Analysis of Similarity (ANOSIM) with 999 permutations was used to compare dissimilarities between samples in different locations based on the Bray-Curtis distance in R package. Kruskal-Wallis test was used to explore variations in genus abundance between different groups. Permutational Multivariate Analysis of Variance (PERMANOVA) based on Bray-Curtis distance was performed to analyze the effects of various factors on the whole fungal diversity. Distance-based redundancy analysis (dbRDA) was conducted to relate the environmental variables with the outdoor culturable fungal community based on Bray-Curtis distance matrice. The envfit function (999 permutations) in the vegan package of R was used to test the statistical significance of environmental factors. The Circos graph was built using Circos-0.69-9 software [58].

## 3. Results

**Figure 2:**
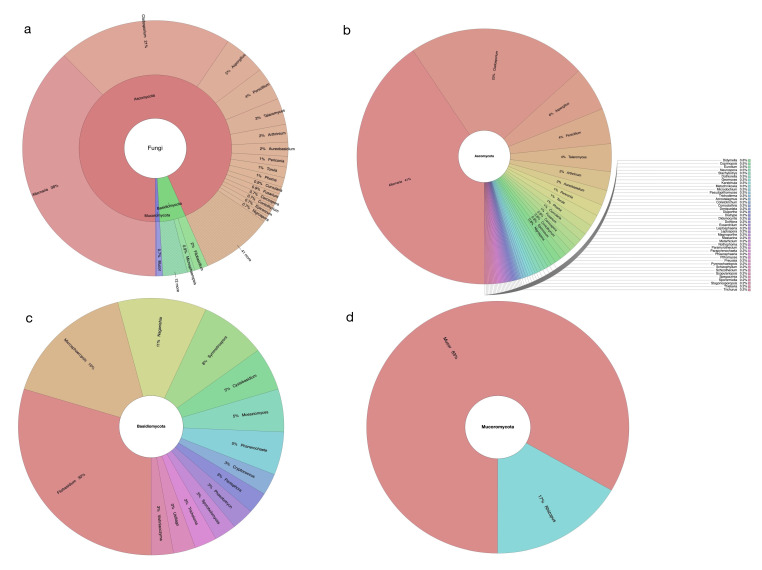
The taxonomic identification and relative abundance of airborne fungi isolated from the building 24. (**a**) Total fungi overview, (**b**) detailed separated view of Ascomycota, (**c**) Basidiomycota, and (**d**) Mucoromycota.

Considering all the analyzed locations in Building 24, in Tianjin University, a total of 667 fungal strains belonging to 160 species and 73 genera of *Ascomycota* (78%), *Basidiomycota* (19%), and *Mucoromycota* (3%) were collected (Figure 2a–d).

The genus *Cladosporium* with 13 identified species, accounting for 8.1% of the total isolated airborne fungal species diversity, was the most diverse taxon, while *Aspergillus* showed the second highest species richness (12 species, 7.5%), followed by *Alternaria* (11 species, 6.9%), and *Penicillium* (8 species, 5%).

Among the dominant fungal species, *Alternaria alternata* (21%) was the most frequent, followed by *A. tenuissima* (12.4%), *Cladosporium cladosporioides* (9.3%), *Aspergillus niger* (2.4%), *C. ramotenellum* (2.1%), *Filobasidium magnum* (1.6%), *Penicillium oxalicum* (1.5%) and *Talaromyces funiculosus* (1.3%) (Supplementary Materials Table S1).

**Figure 3:**
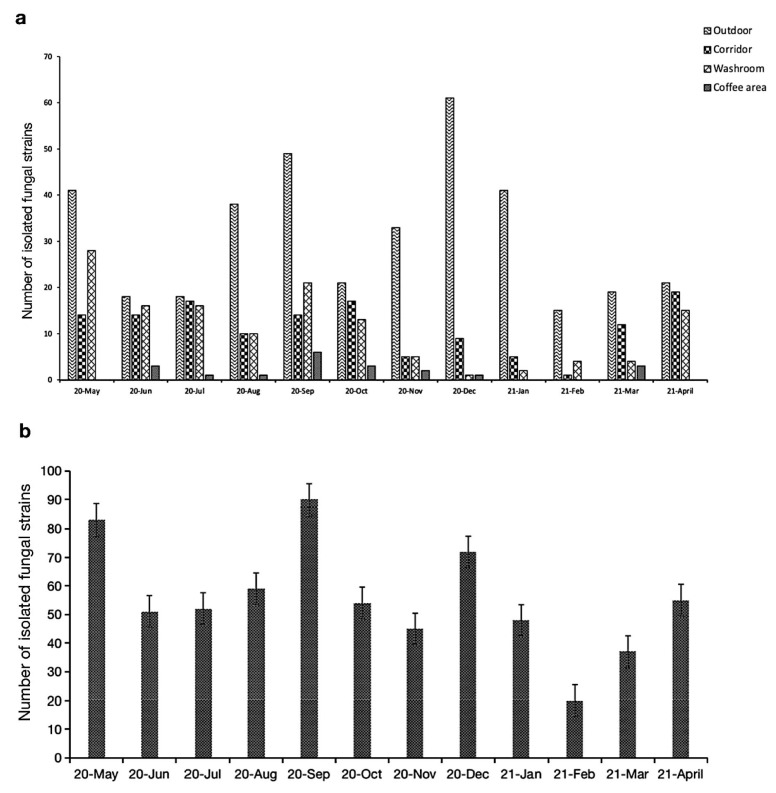
Total strains isolated during the one-year sampling from each location (**a**) and month (**b**).

**Figure 4:**
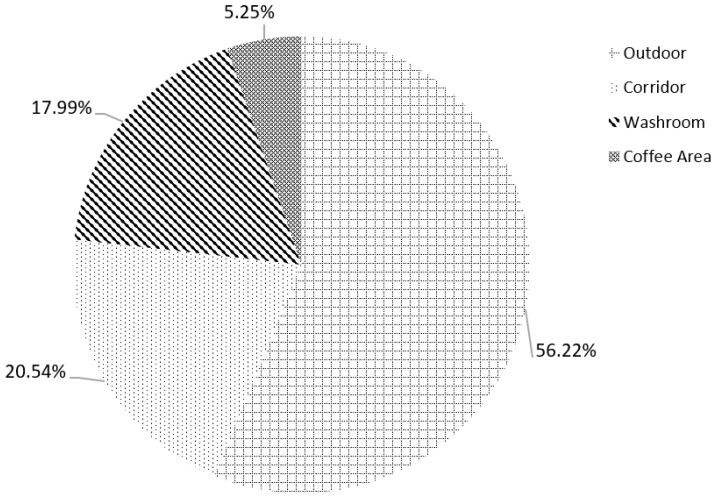
Distribution of fungal strains in different sampling locations.

During the one-year sampling, the highest number of fungal strains was recorded in September (90), while May showed the second highest strain richness (83), followed by December (72). The lowest number of fungi was isolated in February and March, yielding 20 and 38 strains, respectively (Figure 3a,b). Overall, 56.22% of recorded fungal strains were isolated from the outdoor environments, while indoor, 20.54% of fungi were collected from corridor, 17.99% from washroom, and the lowest portion from coffee area (5.25%) (Figure 4).

**Figure 5:**
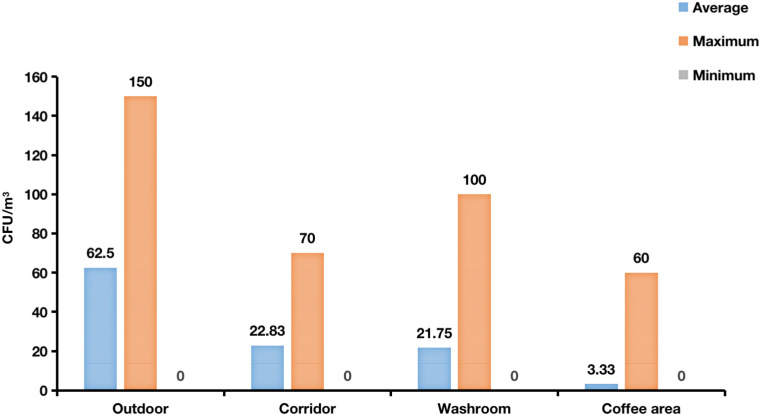
Concentration of airborne fungi in the four sampling environments (CFU/m^3^).

**Figure 6:**
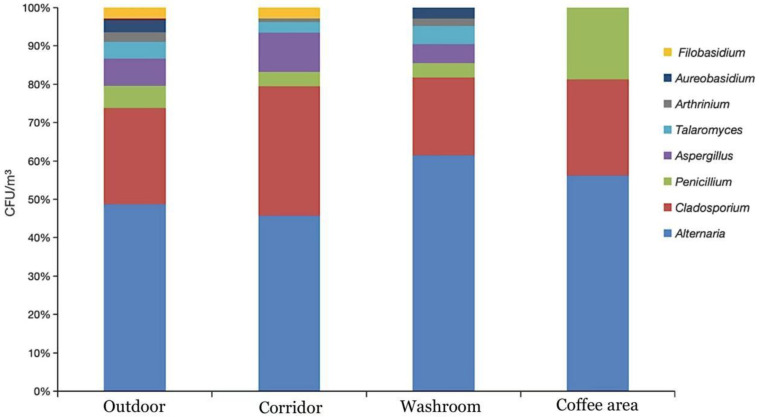
Taxonomic profiles and concentrations of main fungal genera in 4 sampling environments.

The concentrations of airborne fungi in the different analyzed environments ranged from 0 to 150 CFU/m^3^ (Figure 5). The average fungal concentration outdoor was 62.5 CFU/m^3^, whereas for the indoor environments the values were 22.83, 21.75, and 3.33 CFU/m^3^ for corridors, washrooms, and coffee area, respectively (Figure 5). The number of isolated strains of dominant fungal genera *Alternaria*, *Cladosporium*, *Penicillium*, *Aspergillus*, *Talaromyces*, *Arthrinium*, *Aureobasidium*, and *Filobasidium* was higher in outdoor environment than indoor. Washrooms harboured a highest number of *Alternaria*, *Talaromyces*, *Penicillium*, and *Arthrinium* fungi than corridors, which instead showed a higher presence of *Cladosporium*, *Aspergillus*, and *Filobasidium*. The frequency of the dominant fungal genera was relatively low in the coffee area (Table 1; Figure 6).

**Figure 7:**
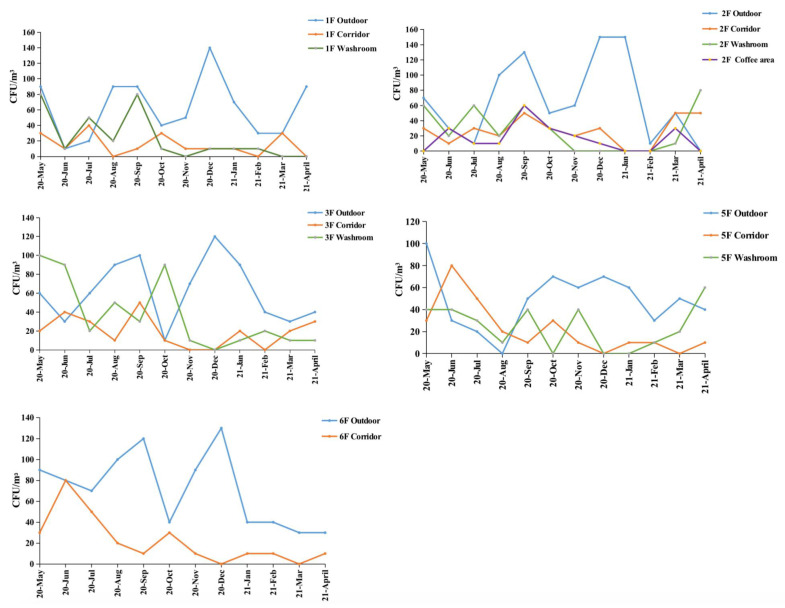
Annual variation of fungal concentration at each floor.

The first floor of the investigated building showed higher outdoor airborne fungal concentrations compared to the other sampling environments during the whole year except July, with the highest level recorded in December (140 CFU/m^3^). The presence of fungal strains in the outdoor air reached a peak in December also at second floor (150 CFU/m^3^), third floor (120 CFU/m^3^), and sixth floor (130 CFU/m^3^). The fungal concentration at second floor was relatively stable in the corridors and coffee area compared to the outdoor environment, where it varied from 0 to 60 CFU/m^3^. At third floor, fungal concentration in washroom area showed a significant variation over the sampling period. It reached the top value of 100 CFU/m^3^ in May and dropped down to 20 CFU/m^3^ two months later, then it increased again to 90 CFU/m^3^ in October and decreased to 10 CFU/m^3^ the next month. At fifth floor, from October to March, the fungal concentration in corridor and washroom, ranging from 0 to 40 CFU/m^3^, was considerably lower than outdoor (30–70 CFU/m^3^). At sixth floor, the outdoor fungal concentration was higher than indoor during the whole year of study, except in October and April (Figure 7).

The Shannon index (H’) values for fungal diversity in different sampling environment in each month are reported in Table 2. Airborne fungal diversity in outdoor environments, showed a peak at 4.46 in December, while the Shannon index was only 0.33 in June. Concerning the indoor environments, the highest Shannon index for corridors was measured in April (3.471), and the lowest (0) in February. For washrooms, the analyzed fungal diversity reached a maximum in April (3.325) and a minimum in December (0), while the coffee area showed very low Shannon index values, with the exception of September (2.59).

**Figure 8:**
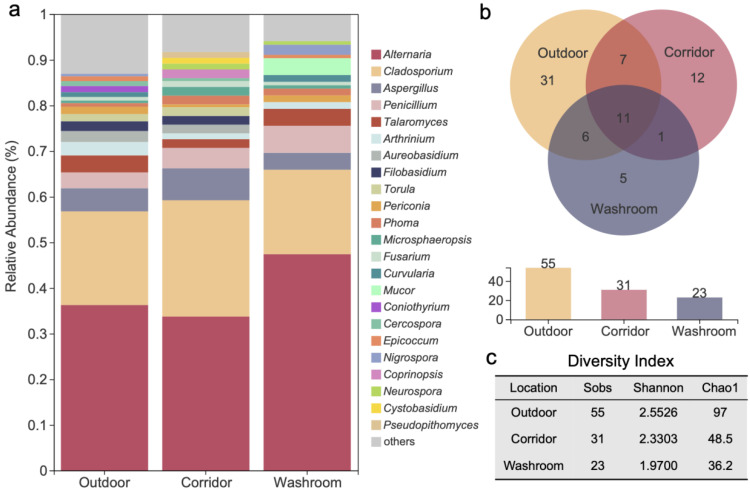
Genus-level fungal diversity and distribution in three sampling locations. (**a**) Relative abundances of dominant fungal genera; (**b**) the Venn diagram displaying the distribution of shared and unique genera; (**c**) diversity indexes (Sobs, Shannon and Chao1) data of fungal communities.

In terms of fungal strain relative abundance, *Alternaria* (38.57%), *Cladosporium* (21.49%), *Aspergillus* (5.34%), *Penicillium* (4.27%), *Talaromyces* (3.20%) and *Arthrinium* (2.29%) were the most abundant fungal genera in the studied teaching building. The genera with low relative abundances (<1%) were merged as “others” in the bar plot (Figure 8a). Among all the 73 detected fungal genera, only 11 were commonly shared by the three different sampling environments in the one-year survey (Figure 8b), including the above mentioned 6 dominant genera, as well as *Phoma*, *Periconia*, *Fusarium*, *Microsphaeropsis*, and *Naganishia* (Supplementary Materials Table S2). Genera richness, Shannon and Chao1 indexes were calculated for outdoor, corridor and washroom fungal communities (Figure 8c). The outdoor environment of the teaching building contained more diverse and richer fungal communities than the corridor and washroom environments, as shown by the significantly higher α-diversity indexes (Figure 8c).

**Figure 9:**
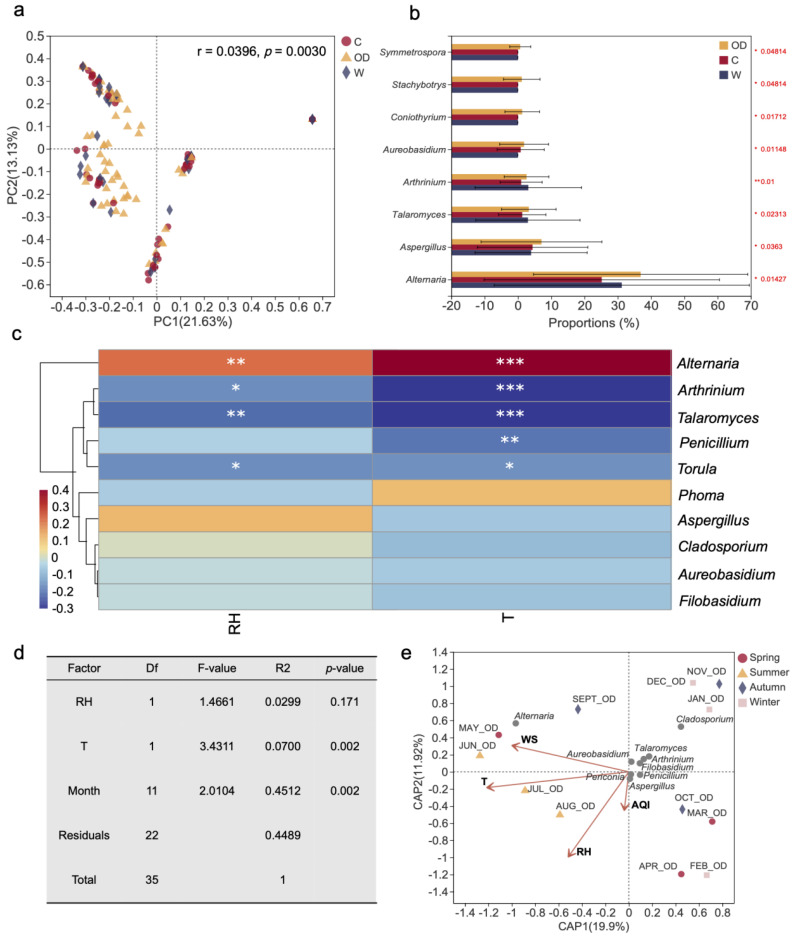
Variance between fungal communities in three sampling locations and effects of environmental factors on the airborne fungal diversity. (**a**) Principal coordinate analysis plot of fungal communities in the sampling building based on Bray–Curtis distance. The r and *p*-value of the analysis of similarity were shown in the figure (*p* < 0.01). (**b**) Comparison of the significantly different genera in the three analyzed locations according to abundances. Differences in the genera as evaluated by Kruskal-Wallis test (**c**) Correlation heat map of the top ten genera and environmental factors. Different colors infer to Spearman’s rank correlation coefficients (r). (**d**) Effects of month, RH and T on whole building fungal community based on permutational multivariate analysis of variance. (**e**) Distance-based redundancy analysis of the outdoor fungal communities with symbols coded by seasons and top ten fungal genera. In the sub-figures, OD, C and W refer to “Outdoor”, “Corridor” and “Washroom”, while T, RH, WS and AQI refer to “temperature”, “relative humidity”, “wind speed” and “air quality index”, respectively. The value *p* is indicated as: * *p* < 0.05, ** *p* < 0.01, *** *p* < 0.001.

The variations of fungal genera were analyzed by principal co-ordinate analysis (PCoA). Fungal communities in the outdoor, corridor and washroom of the teaching building air were clearly distinguished based on Bray–Curtis distance (ANOSIM *p* = 0.003) (Figure 9a). The abundances of the fungal genera *Alternaria*, *Aspergillus*, *Talaromyces*, *Arthrinium*, *Aureobasidium*, *Coniothyrium*, *Stachybotrys* and *Symmetrospora* occupied significant higher proportions of the outdoor environment (Figure 9b). The recorded environmental parameters (Supplementary Materials Table S3) showed varying effects on different fungal genera and on the whole fungal community detected in the analyzed building. Among the top twenty genera detected, *Alternaria* revealed significant positive correlations with temperature, while *Arthrinium*, *Talaromyces*, *Penicillium* and *Torula* were negatively correlated with temperature (Figure 9c; Supplementary Materials Table S4). *Alternaria* also showed a strong, positive correlation with relative humidity, which was instead negatively correlated with *Arthrinium*, *Talaromyces* and *Torula* (Figure 9c; Supplementary Materials Table S4). In particular, the correlations between *Alternaria* (positive), *Arthrinium* (negative) and *Talaromyces* (negative) abundance and temperature were extremely significant (*p* < 0.001). Sampling month and temperature showed significant impacts on the airborne fungal community with a *p*-value of 0.002 (Figure 9d). On the contrary, the influence of relative humidity on the detected fungal diversity was low (*p*-value = 0.171; Figure 9d). For the analyzed outdoor environments, dbRDA based on fungal genera revealed that temperature (r^2^ = 0.9033, *p* = 0.001), relative humidity (r^2^ = 0.7607, *p* = 0.006) and wind speed (r^2^ = 0.6010, *p* = 0.020) were highly correlated with fungal community composition (Figure 9e), while the influence of air quality index was not significant (r^2^ = 0.1082, *p* = 0.610) (Figure 9e). Besides, temperature caused a relatively higher influence on the summer outdoor fungal community of the investigated building compared to the other sampling seasons, which is represented by the closer distance between the summer samples (JUN_OD, JUL_OD and AUG_OD) and the temperature arrow in the Figure 9e. On the contrary, during the winter season, the airborne fungal diversity was not much affected by the various recorded parameters, as shown in Figure 9e, where the winter samples (DEC_OD, JAN_OD) are very far from the environmental factor arrows. Outdoor *Alternaria* and *Cladosporium* abundances were much more affected by the analyzed environmental factors than in other genera (Figure 9e).

**Figure 10:**
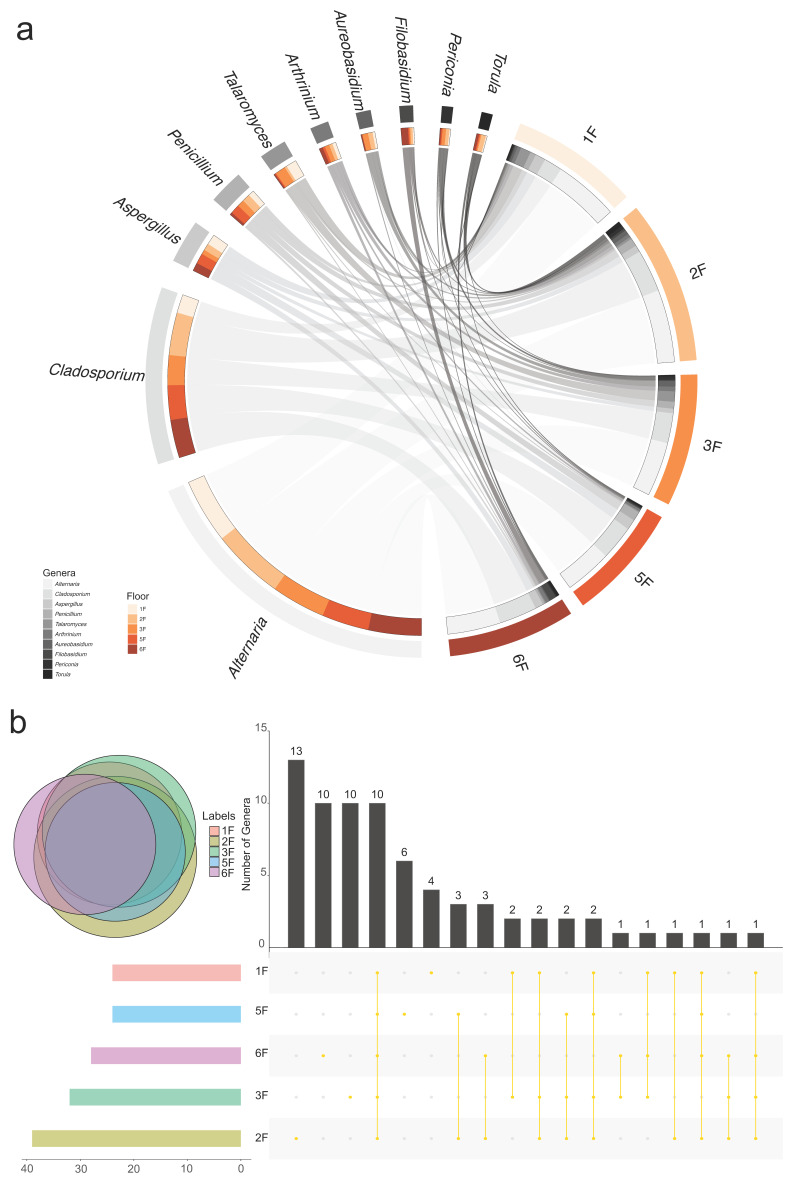
Fungal diversity and distribution in five sampling floors. (**a**) Distribution of top ten fungal genera in each floor visualized by Circos, the width of the bars indicates the relative abundance. (**b**) Number of common and unique genera detected between floors.

Looking at the fungal community structure in the five sampling floors, *Alternaria* and *Cladosporium* were the dominant genera in each floor (Figure 10a; Supplementary Materials Table S5). However, the relative abundance of *Alternaria* fungi in the first floor of the studied building was significantly higher than in the other floors, where by contrast, *Cladosporium* was much more abundant. The genus *Filobasidium*, the sole Basidiomycetous taxon recorded among the top ten genera, was mostly detected in the sixth floor (Figure 10a), while *Aureobasidium* strains were isolated from all the analyzed floors, except the fifth floor (Figure 10a; Supplementary Materials Table S5). The genus *Naganishia* was present in the first three floors, while it was not observed in the fifth and sixth floors (Supplementary Materials Table S2). The most diverse fungal community was detected from the second floor, which harboured the highest total number of genera (Figure 10b), and the highest number of unique genera (13), including *Cystobasidium*, *Diatrype*, *Spegazzinia* and *Tricholoma*, which were only isolated from the coffee area air samples. Ten genera were commonly shared by all five floors (Figure 10b; Supplementary Materials Table S2), including *Filobasidium*, *Periconia*, *Fusarium*, *Torula*, together with the 6 genera above mentioned as the most abundant taxa of the whole study (*Alternaria*, *Cladosporium*, *Aspergillus*, *Penicillium*, *Talaromyces*, and *Arthrinium*; Supplementary Materials Table S2).

## 4. Discussion

This is the first study on the cultivable airborne fungal diversity and concentration in a teaching and research building of Tianjin University, a major university area of one of the biggest cities of China. The 15 sampling sites, including outdoor and indoor environments, showed a rich fungal community, which yielded 667 isolated fungal strains belonging to 160 species and 73 genera. The concentration of airborne fungi ranged from 0 to 150 CFU/m^3^, which is much lower than the threshold value of 2500 CFU/m^3^ for total airborne microorganism in Chinese residential and office buildings established by the Ministry of Health (MOH), Ministry of Environmental Protection (MEP), and the General Administration of Quality Supervision, Inspection and Quarantine (AQSIQ) of China in the Indoor Air Quality Standard issued in 2002 [59]. The recorded fungal concentration in all the analyzed environments was also within the limit value of 1000 CFU/m^3^ recommended by the Occupational Safety and Health Administration (OSHA) of United States [60], and the threshold values published by the World Health Organization (WHO) [61]. Previous research carried out in different countries have shown that the concentration of airborne fungi in school buildings varied from 10 to 3000 CFU/m^3^ [40,62,63,64]. The statistical analyses performed in our study demonstrated that sampling month, temperature, relative humidity, and wind speed (*p* = 0.002) had significant impact on the investigated airborne fungal community, diversity and concentration. Previous studies have shown that seasonal variations, types of ventilation, human activities, and other abiotic factors effected the airborne fungal concentrations in the analyzed environments [9,31,32,40,65].

The most abundant fungal genera found in the investigated sites were *Alternaria*, *Cladosporium*, *Aspergillus*, *Penicillium*, *Talaromyces*, and *Arthrinium* (Figure 1 and Figure 7a). Among these dominant genera, *Cladosporium*, *Aspergillus*, and *Penicillium* have been widely reported in school environments in Australia [40], Ethiopia [66], and America [29,67]. *Alternaria* was the dominant fungal genus comprising about 40% of all isolated fungal strains, which is in agreement with a previous study conducted at city level in Tianjin, where *Alternaria* accounted for nearly one fourth of all the isolated airborne fungal strains [9]. Fungi belonging to the genus *Alternaria* are recognized as common plant pathogens, whose abundance in the analyzed air environments may be supported by the large presence of tree vegetation in the Tianjin University campus [68]. Indeed, the phylloplane (leaf surface) availability has been described as an important factor to sustain the presence and growth of parasitic and saprotrophic fungi in urban green areas, and therefore as a source for the dispersal of fungal air spores [69]. Besides, long distance transport of *Alternaria* conidia originating from crops and orchards situated in the surrounding territory of Tianjin could supplement the airborne catch recorded at the investigated site. Fernández-Rodríguez et al., (2015) [70] previously connected the atmospheric concentration of *Alternaria* spores in Badajoz, South-west Spain, to different types of agricultural land in the surrounding areas by analyzing the air mass transport. The presence of *Alternaria* fungi inside the studied building probably originated from outdoor air through natural ventilation, since large-spore fungal genera like *Alternaria* are normally trapped by air filters in the mechanical ventilation and air-conditioning (MVAC) system [71]. Within the genus *Alternaria*, the species *A. alternata* appeared most frequently in the whole study. This species has been described as the prevalent airborne fungal taxon in nasal discharge, with potential vigorous immunologic activities in nasal epithelial cells [72]. In addition, *A. alternata* is mainly related to the respiratory diseases which are mediated by the induction of immunoglobulin E (IgE) [9,73]. The spores of this fungal species are considered to be one of the most abundant and potent sensitizer allergens in the air [73]. *Cladosporium*, the second most common genus in the analyzed university building, has previously shown a dominant presence in Tianjin city outdoor environments, including both green areas and sites characterized by intense car traffic [9]. Spores produced by *Cladosporium* species are known to be abundantly spread in the air worldwide, being the dominant airborne spores in temperate climates [74]. They are primarily outdoor fungal taxa with melanized cell walls, commonly colonizing plant tissues [75], which are likely to have settled in dust in the analyzed building from outdoor air. However, previous studies suggested that *Cladosporium* fungal presence in the indoor environments could be influenced by CO_2_ concentration and number of occupants [76,77]. It is possible that the aforementioned parameters, not measured in our work, played a role in sustaining the presence of *Cladosporium* in the studied Tianjin University building through internal sources. Further analyses would be needed to test this hypothesis. The genus *Cladosporium* previously included more than 772 taxa [30], but only 170 have been recently recognized as distinct species [78], with *C. cladosporioides*, *C. herbarum*, and *C. sphaerospermum* being considered the three major species complexes [79]. Among them, *C. cladosporioides* has been found to cause Phaeohyphomycotic dermatitis in giant panda [80]. Castro et al., (2013) have reported that a 27-year-old non-smoking and immunocompetent female chemical engineer who worked at a cork company in Portugal suffered a pulmonary infection caused by *C. cladosporioides* [81]. *Aspergillus* was the third highest diverse fungal genus found in our sampling environments. *Aspergillus* fungi are common in soil ecosystems, do not have cell wall melanin, and their airborne behavior is mostly associated to indoor environments [82]. The dominant presence of *Aspergillus* species in the indoor aerosol is usually attributed to environmental conditions that support indoor fungal growth [76,83]. This genus, which comprises over 180 species [84], can induce infections, mainly through the respiratory tract, in immunocompromised hosts and in people with underlying pulmonary disease [85]. Moreover, it has been demonstrated that *Aspergillus* fungi can invade other tissues such as skin, central nervous system, eyes, and nails [85,86,87,88,89]. In the United Kingdom the spores of *Aspergillus* species have been reported to be dominant in the air during autumn and winter seasons [90]. The genus *Penicillium*, which showed the fourth highest diversity in our study, has been previously associated with increased peak expiratory flow variability in asthmatic children [91]. *Penicillium* species are known to be prevalent airborne fungi in various indoor environments [26]. It has been demonstrated that inhalation of a considerable number of *Penicillium* spores can induce immediate and late asthma in sensitive individuals [26].

Our results showed that the concentrations and total number of airborne fungal strains outdoor were significantly higher than indoor over the analyzed 12 months. Moreover, higher α-diversity indexes showed that the airborne fungal communities were more diverse outdoor than indoor. This observation is in agreement with previous studies in which the annual concentrations of airborne fungi outdoor were all higher than in the different analyzed indoor environments [44,49,92]. Similarly, a previous study focusing on an office building in Korea revealed that the airborne fungal concentration indoor was consistently lower than outdoor over an 18 month-period [93]. Madsen et al., (2012) sampled airborne inhalable dust in five Danish homes throughout the four seasons of one year and showed that the concentration of fungi in the analyzed samples was significantly higher outdoor than indoor in spring, summer, and autumn, while there was minimal difference in winter [49]. Wu et al., (2020) investigated the contamination level of airborne fungi in various rooms in the university of Xi’an, China, during autumn and winter, and found that indoor/outdoor airborne fungal concentration ratios were all below 1.0 [44]. Sautour et al., (2009) found that fungal community dynamic was very similar in various explored units of a public building, but markedly different from that observed in outdoor air [94]. The lower indoor airborne fungal concentration rates compared to the outdoor environment registered in our study could be caused by the mechanical ventilation which can prevent intrusion in indoor areas of a large fraction of fungi present outdoor, while a partial penetration of outdoor airborne fungi would be allowed through the natural ventilation via doors and windows [92]. The ventilation status of indoor environments (natural vs mechanical) can play a crucial role in defining the fungal diversity and concentration. Accurate maintenance and fine-tuned operating conditions of the MVAC, combined with the use of suitable air filters may prevent indoor contamination by outdoor microbial sources [95]. A study carried out by Zhou et al., (2021) in Beijing, China, during the winter season, showed that Shannon indices of indoor airborne fungal diversity were lower than outdoor, which was explained with the presence of both limited fungal emission sources and various chemical pollutants such as CO_2_, CO, formaldehyde, methane in the studied indoor areas [96]. However, Lee et al., (2006) found opposite results in a research work analyzing six single-family homes in Cincinnati metropolitan area (USA), where the diversity of indoor airborne fungi was much greater than that measured outdoor, which indicated that indoor environments provided more favorable conditions for survival of airborne fungal spores [97]. Further studies are needed to clarify the relationship between indoor and outdoor fungal communities and to disentangle the roles that different environmental parameters play in shaping the diversity and concentration of airborne fungi in adjacent indoor and outdoor areas.

Only 11 genera (*Alternaria*, *Arthrinium, Aspergillus, Cladosporium*, *Fusarium*, *Microsphaeropsis, Naganishia, Penicillium*, *Periconia, Phoma*, and *Talaromyces*) were shared by three different investigated sampling environments in our study (Figure 8b). Principal coordinate analysis showed the presence of clearly distinct fungal communities in the outdoor, corridor and washroom areas (Figure 9a). In the analyzed outdoor environments, 55 different fungal genera were found in total, which was more than twice higher than in the corridors (21) and washrooms (23). This difference in the analyzed airborne fungal communities could be caused by outdoor temperature and relative humidity, which were dramatically higher than indoor, especially in summer. It is worth noting that high temperature and rich humidity have been previously reported to boost microbial growth in summer [98]. Moreover, it is possible that the wind, which is known to enhance the spread and diversity of airborne fungi [99], produced a stronger effect on the analyzed outdoor environment, which was attenuated indoor, thus creating a difference between indoor and outdoor fungal communities.

Previous studies have shown that a number of environmental factors, such as temperature, relative humidity, and wind speed may have great effect on the diversity and community structure of airborne fungi in various sampling environments. The statistical analyses performed in our study demonstrated that sampling month and temperature (*p* = 0.002) had significant impact on the airborne fungal communities, among the analyzed environmental parameters (Figure 9d). This is consistent with previous research, which have shown that seasonal variation of the environment may affect airborne fungal community diversity and concentration. For example, summer season has been found to show higher airborne fungal diversity related to higher temperature and relative humidity, whereas winter has been reported as the season with the lowest presence of fungi in the air [2]. However, different fungal genera may have different adaptability in the same environments. In our investigated building, the fungal genus *Alternaria* revealed significant positive correlations with temperature and relative humidity, while *Arthrinium*, *Talaromyces* and *Torula* showed negative correlation with the above-mentioned environmental factors. Previous studies reported that the concentration of *Alternaria* fungi was significantly higher from May to July compared to other months, while the total fungal concentration in autumn was higher than in winter [28,45]. It has been demonstrated that people trampling leaves outside in autumn may increase the likelihood of bringing plant debris in buildings, which in turn may support a higher diversity of airborne fungi indoor [28,45]. Our study confirmed the influence of seasons on airborne fugal communities, showing that the concentration of fungi in the analyzed environments was higher in summer and autumn than in winter, being the temperature seasonal variation a crucial factor to determine the observed differences in the fungal community during the one-year sampling. We found that the correlation between the analyzed environmental variables, including temperature (r^2^ = 0.9033, *p* = 0.001) and relative humidity (r^2^ = 0.7607, *p* = 0.006), and the fungal community composition was higher in outdoor environment, which is in agreement with previous researches that have described temperature and humidity playing key roles in the outdoor airborne fungal distribution and diversity [2,100]. The airborne fungal community outdoor the studied building was more significantly affected by the temperature during summer, whereas in winter the effect of each measured environmental parameter on the outdoor airborne fungi was not significant (Figure 9e). Further studies are needed to clarify the seasonal variation in airborne fungal community diversity and structure, and to better understand the role of different environmental parameters on each fungal taxon constituting a certain community over varying seasons. In this respect, we found that the effect of outdoor environmental parameters was more pronounced on *Alternaria* and *Cladosporium* than on other fungal genera, which is in agreement with the results from Anees-Hill et al., (2021), who observed a stronger effect of the seasonal variation of environmental parameters on the presence and concentration of *Alternaria* and *Cladosporium,* compared to the average effect on the total fungal community [65]. Numerous studies have shown that meteorological variables are associated with fungal seasonality [40,44,47]. The experimental results of our study showed that the wind speed had close relationship with the diversity and concentrations of airborne fungi, which may be due to the facts that wind enhances the dispersal of fungal spores in the air by carrying them away from the fungal structures, such as fruit bodies and conidiophores, growing in the soil, on the plant surface, and other various environmental sources [101]. The majority of fungal spores require at least 5 m s^−1^ wind speed to be detached from conidiophores [102], while for some fungi, such as *Alternaria* species, spore liberation requires stronger wind, because the conidia are firmly attached to the conidiophores [103]. Moreover, lower wind speed or stagnant air may lead to accumulation of pollutants in the air which could negatively affect the airborne fungal survivability [104]. Our findings are in agreement with a previous study performed by Nageen et al., (2020) in Tianjin, which showed that the wind speed was the most important factor affecting the fungal community in different outdoor environments located in the four main city districts [9]. Similar results were found in a study on the impact of environmental factors on outdoor airborne fungi on the terrace of the Faculty of Science at the University of Extremadura (Badajoz, Spain) situated at 16 m above ground level [70]. We found that the influence of the air quality index (AQI PM2.5) on the recorded fungal diversity was not significant (r^2^ = 0.1082, *p* = 0.610) (Figure 9e), which is in agreement with the results found by Tang et al., (2020) who characterized the fungal assemblages in PM2.5 samples collected in a nursery pig house during different seasons [105]. On the contrary, Dong et al., (2016) explored the composition of airborne fungi in PM2.5 in suburban Beijing and discovered that the species richness and diversity of fungal communities was falling with the aggravation of air pollution [53]. In a study performed in the coastal area of Qingdao, China, it was shown that PM2.5, SO_2_, NO_2_, CO and the air quality index (AQI) had significant positive correlations with the airborne microbe concentration during hazy days [106]. However, the effect of air pollution on airborne fungal diversity deserves further attention to be defined.

Our study suggested that variations in the relative abundance of major airborne fungal taxa at different heights above-ground could lead to a difference in the community structure, as showed in the Circos graph, representing the distribution of top ten fungal genera in each floor (Figure 10a). Although *Alternaria* and *Cladosporium* were constantly the most dominant genera at each floor (Figure 10a; Supplementary Materials Table S5), the relative abundance of each airborne fungal genus varied significantly between different floors. In the first floor of the investigated building, the relative abundance of *Alternaria* was significantly higher than in other floors. On the contrary, the relative abundance of the fungal genus *Cladosporium* was higher in other floors than in the first floor. The warmer average temperature registered in the first-floor environment could be responsible for the higher relative abundance of *Alternaria*, as well as for the lower presence of *Cladosporium*, compared to the other analyzed floors. In fact, the latter two fungal genera were shown to be affected in an opposite way by the temperature in the overall study (Figure 9c; Supplementary Materials Table S4). Besides, we observed that the first floor of the studied building was characterized by the highest flow of people and the best ventilation system with more entrances and exits, which may influence the diversity and concentration of airborne fungi via environmental changes. The different distribution of *Alternaria* and *Cladosporium* fungi could also be explained with the difference in their spore size. In fact, the multicellular large-sized conidia of *Alternaria* are likely to be more abundant at lower heights due to their low buoyancy, whereas *Cladosporium* smaller sized spores could show a general trend of concentration at higher floors due to their easier dispersal at greater heights [107]. Li et al., (2010) [108], in a study performed in Shenzhen University, China, recorded a higher concentration of fungi with larger spore size at 10 m height, while fungi with smaller spores, such as *Cladosporium*, were more concentrated at 70 m. Fungi belonging to the genus *Filobasidium* were mostly detected in the sixth floor (Figure 10a), a rooftop rarely visited by people, while their relative abundance was lower in other floors with intense human activities, especially the first and the third floors, where it was lower than 1.0% (Supplementary Materials Table S5). *Filobasidium* is a basidiomycetous yeast genus that commonly colonizes plant material [109]. The presence of this fungal taxon at the rooftop of the investigated building could be related to outdoor dust particle deposition and rain accumulation. Kolek et al., (2021) compared the fungal diversity between ground-level and rooftop-level (12 m above ground) in two residential buildings in Augsburg, Germany, showing that sampling heights played an important role in affecting the presence of particular fungal taxa, which was confirmed by our results [110]. However, the presence and concentration of some fungal taxa in our studied building could be influenced by the level of human activity, which needs to be determined with further studies focusing on this specific factor. *Aureobasidium* strains were isolated from all the analyzed floors, except the fifth floor, showing their highest relative abundance (4.171%) at third floor (Figure 10a; Supplementary Materials Table S5). This fungal genus was reported to cause subcutaneous phaeohyphomycosis in an immunocompetent carpenter in a study conducted in Morocco [111]. The hazardous genus *Naganishia*, which was found to cause Otomycosis [112] and superficial skin infection [113] in previous studies performed in Iran, was found in first floor corridor, second floor washroom and third floor outdoor environment of the analyzed building, while it was not observed in the fifth and sixth floors (Supplementary Materials Table S2). The presence of *Naganishia* in the studied university building confirmed the previous report of this fungal genus in Tianjin by Nageen et al., (2021) [9]. In the latter study, *Naganishia* was the third most abundant fungal genus detected at city level. *Naganishia* can be considered a peculiar presence in Tianjin outdoor and indoor environments, as this genus was not reported in any other study on airborne fungi conducted in China before.

Our study suggested that variations in the relative abundance of major airborne fungal taxa at different heights above-ground could lead to a difference in the community structure, as showed in the Circos graph, representing the distribution of top ten fungal genera in each floor (Figure 10a). Although *Alternaria* and *Cladosporium* were constantly the most dominant genera at each floor (Figure 10a; Supplementary Materials Table S5), the relative abundance of each airborne fungal genus varied significantly between different floors. In the first floor of the investigated building, the relative abundance of *Alternaria* was significantly higher than in other floors. On the contrary, the relative abundance of the fungal genus *Cladosporium* was higher in other floors than in the first floor. The warmer average temperature registered in the first-floor environment could be responsible for the higher relative abundance of *Alternaria*, as well as for the lower presence of *Cladosporium*, compared to the other analyzed floors. In fact, the latter two fungal genera were shown to be affected in an opposite way by the temperature in the overall study (Figure 9c; Supplementary Materials Table S4). Besides, we observed that the first floor of the studied building was characterized by the highest flow of people and the best ventilation system with more entrances and exits, which may influence the diversity and concentration of airborne fungi via environmental changes. The different distribution of *Alternaria* and *Cladosporium* fungi could also be explained with the difference in their spore size. In fact, the multicellular large-sized conidia of *Alternaria* are likely to be more abundant at lower heights due to their low buoyancy, whereas *Cladosporium* smaller sized spores could show a general trend of concentration at higher floors due to their easier dispersal at greater heights [107]. Li et al., (2010) [108], in a study performed in Shenzhen University, China, recorded a higher concentration of fungi with larger spore size at 10 m height, while fungi with smaller spores, such as *Cladosporium*, were more concentrated at 70 m. Fungi belonging to the genus *Filobasidium* were mostly detected in the sixth floor (Figure 10a), a rooftop rarely visited by people, while their relative abundance was lower in other floors with intense human activities, especially the first and the third floors, where it was lower than 1.0% (Supplementary Materials Table S5). *Filobasidium* is a basidiomycetous yeast genus that commonly colonizes plant material [109]. The presence of this fungal taxon at the rooftop of the investigated building could be related to outdoor dust particle deposition and rain accumulation. Kolek et al., (2021) compared the fungal diversity between ground-level and rooftop-level (12 m above ground) in two residential buildings in Augsburg, Germany, showing that sampling heights played an important role in affecting the presence of particular fungal taxa, which was confirmed by our results [110]. However, the presence and concentration of some fungal taxa in our studied building could be influenced by the level of human activity, which needs to be determined with further studies focusing on this specific factor. *Aureobasidium* strains were isolated from all the analyzed floors, except the fifth floor, showing their highest relative abundance (4.171%) at third floor (Figure 10a; Supplementary Materials Table S5). This fungal genus was reported to cause subcutaneous phaeohyphomycosis in an immunocompetent carpenter in a study conducted in Morocco [111]. The hazardous genus *Naganishia*, which was found to cause Otomycosis [112] and superficial skin infection [113] in previous studies performed in Iran, was found in first floor corridor, second floor washroom and third floor outdoor environment of the analyzed building, while it was not observed in the fifth and sixth floors (Supplementary Materials Table S2). The presence of *Naganishia* in the studied university building confirmed the previous report of this fungal genus in Tianjin by Nageen et al., (2021) [9]. In the latter study, *Naganishia* was the third most abundant fungal genus detected at city level. *Naganishia* can be considered a peculiar presence in Tianjin outdoor and indoor environments, as this genus was not reported in any other study on airborne fungi conducted in China before.

To sum up, the results of our study revealed that there were remarkable fluctuations in the airborne fungal diversity and concentration of indoor and outdoor sampling environment in different seasons. Environmental parameters, such as temperature, relative humidity, and wind speed, fluctuating with the seasonal variation were key elements influencing the airborne fungal diversity at each studied sampling site. In addition, the different heights of floors also affected the distribution and diversity of airborne fungi in both indoor and outdoor environments of the analyzed building.

## 5. Conclusions

Our study represents the first systematical survey on the airborne fungal diversity in a building of Tianjin University. The data collected during the one year long sampling allowed us to clarify the seasonal dynamics of the investigated fungal communities and to correlate the diversity and concentration of recorded fungi with environmental factors. Our results showed that temperature, relative humidity and wind speed, changing with seasonal variation, significantly affected the composition of the analyzed airborne fungal communities, while the influence of the air quality index (PM2.5) was not significant. The height above ground level also played a key role in affecting the airborne fungal community structure at different floors of the studied building, partly probably due to the different aerodynamic behavior of spores produced by the recorded fungal species, partly depending on the ventilation status, which could produce a different level of indoor fungal contamination from outdoor sources due to shifting conditions between mechanical and natural ventilation. Overall, the rich fungal diversity characterizing the studied air environments showed concentration values that were always lower than thresholds recommended in air quality standards. The dominant presence of fungal genera such as *Alternaria, Cladosporium, Aspergillus,* and *Penicillium*, which are known for their potentially harmful effects on human health, deserves further attention and monitoring for prevention of airborne fungi associated diseases. The results of this study provide valuable information on the factors shaping the indoor and outdoor fungal concentrations and community structure in university buildings and may represent an important reference for air quality monitoring, microbial pollution control, and airborne diseases prevention in environments characterized by high human activity.

## Figures and Tables

**Table 1:** The distributions of main fungal genera in 4 sampling environments.

Fungal Genera	Outdoor	Corridor	Washroom	Coffee Area
*Alternaria*	136	49	64	4
*Cladosporium*	78	36	24	4
*Penicillium*	13	4	8	3
*Aspergillus*	19	11	5	0
*Talaromyces*	13	3	6	0
*Arthrinium*	11	2	2	0
*Aureobasidium*	9	0	3	0
*Filobasidium*	8	3	0	0

**Table 2:** Shannon index of diversity of fungal communities in different sampling environments throughout the year.

Shannon Index (H’)												
Sampling Environments	1-May	1-Jun	1-Jul	1-Aug	1-Sep	1-Oct	1-Nov	1-Dec	1-Jan	1-Feb	1-Mar	1-Apr
Outdoor	1.253	0.33	2.131	3.237	3.575	3.366	3.771	4.459	4.336	2.923	3.735	3.746
Corridor	2.807	2.264	2.524	2.059	2.02	2.778	2.322	2.725	2.322	0	2.585	3.471
Washroom	2.429	1.921	2.18	2.912	2.521	3.18	1.858	0	1	2	1.5	3.325
Coffee Area	0	1.585	0	0	2.585	1.585	1	0	0	0	0.918	0

## Data Availability

The fungal DNA sequences amplified during this study are available at GenBank under accessions ON208139—ON208805.

## Supplementary Materials

The following supporting information can be downloaded at: https://www.mdpi.com/article/10.3390/jof8050431/s1, Table S1: Isolated airborne fungal species and number of strains. Table S2: Total number of fungal colonies isolated from each location from May 2020 to April 2021. Table S3: Environmental factors recorded in each sampling site at the time of sampling. Table S4: Correlation analysis between fungal abundance and environmental parameters. Table S5: Relative abundances of top ten genera in five sampling floors.
