# Supplementary material for: Environmental Factors Affecting Diversity, Structure, and Temporal Variation of Airborne Fungal Communities in a Research and Teaching Building of Tianjin University, China
Source: J Fungi (Basel). 2022 Apr 22;8(5):431. doi: 10.3390/jof8050431 (PMC9144611; doi:10.3390/jof8050431)
Supplement: Supplementary file 1 [file jof-08-00431-s001.zip › jof-1679956-supplementary.pdf]

Environmental Factors Affecting Diversity, Structure, and Temporal Variation of Airborne Fungal Communities in a Research and Teaching Building of Tianjin University, China

Yixuan Lu †, Xiao Wang †, Lucineidy C. S. de S. Almeida and Lorenzo Pecoraro *

School of Pharmaceutical Science and Technology, Tianjin University, 92 Weijin Road, Tianjin 300072, China; luyixuann2022@163.com (Y.L.); wang_xiao1996@163.com (X.W.); lu.kaili551@gmail.com (L.C.S.d.S.A.)

* Correspondence: lorenzo.pecoraro@tju.edu.cn

† Co-first author.

Table S1. Isolated airborne fungal species and number of strains.

Fungal species	May-20	June-20	July-20	Aug.-20	Sep.-20	Oct.-20	Nov.-20	Dec.-20	Jan.-21	Feb.-21	Mar.-21	Apr.-21	Total
Acrostalagmus sp.									1				1
Alternaria alternata	46	28	16	13	5	4	3	12	3	1	4	5	140
Alternaria arborescens	1												1
Alternaria destruens	2												2
Alternaria doliconidium	1												1
Alternaria infectoria					2								2
Alternaria longipes							1						1
Alternaria pharbitidis		1											1
Alternaria quercus			1										1
Alternaria sp.	3	2	2		5	1			6		1		20
Alternaria tamaricis			1										1
Alternaria tenuissima	7	2	17	11	24	6	2			2	6	6	83
Arthrinium malaysianum								1					1
Arthrinium marii								2					2

Arthrinium phaeospermum								1					1
Arthrinium piptatheri							1						1
Arthrinium saccharicola				1	1		1	1					4
Arthrinium sp.							2	1	1	2			6
Aspergillus awamor				1									1
Aspergillus costaricensis						1							1
Aspergillus niger	5	4		1				1		4	1		16
Aspergillus nomius	2												2
Aspergillus ochraceus							1						1
Aspergillus protuberus						1		1					2
Aspergillus pseudoglaucus						1							1
Aspergillus sp.										2			2
Aspergillus stellatus										1			1
Aspergillus sydowii						1							1
Aspergillus tubingensis				2	1			2					5
Aspergillus versicolor		1										1	2
Aureobasidium melanogenum					1								1
Aureobasidium namibiae							1						1
Aureobasidium proteae								1					1
Aureobasidium pullulans				1	4								5
Aureobasidium sp.		1					2	1					4
Cercospora arctii-ambrosiae								1					1
Cercospora canescens				1									1
Cercospora nicotianae					2				1				3
Cladosporium anthropophilum			2	2	1	3			1				9
Cladosporium sinuosum												1	1
Cladosporium austroafricanum					1			2				1	4
Cladosporium cladosporioides	1			2	20	12	7	7	6	2	3	2	62
Cladosporium colombiae									1			1	2
Cladosporium delicatulum			3										3
Cladosporium gossypiicola			2			2							4

Cladosporium halotolerans					1								1
Cladosporium oxysporum			1										1
Cladosporium ramotenellum	2		1		2			5				4	14
Cladosporium sp.		1	1		2		3	9		2	2	7	27
Cladosporium sphaerospermum					1								1
Cladosporium tenuissimum			4	1	1	1	3	1	1			1	13
Colletotrichum gloeosporioides						1							1
Coniothyrium pyrinum							3						3
Coniothyrium sp.					1								1
Comoclathris sp.												1	1
Coprinopsis atramentaria				1			1					1	3
Coniothyrium aleuritis												1	1
Cryptococcus flavescens								1					1
Curvularia sp.				2									2
Curvularia lunata				2									2
Curvularia intermedia					1								1
Curvularia verruculosa				1									1
Cystobasidium sp.											2		2
Deniquelata quercina						1							1
Diaporthe sp.				1									1
Diatrype stigma				1									1
Didymella subherbarum	1												1
Didymella macrostoma				1									1
Didymocyrtis brachylaenae											1		1
Didymella pedeiae												2	2
Dothiorella gregaria												2	2
Dothiora europaea									1				1
Epicoccum nigrum					1			1					2
Epicoccum layuense											1		1
Epicoccum sp.				1	1								2
Eurotium sp.								1	1			1	3

Exserohilum rostratum				1									1
Filobasidium magnum		1	1		1			5	1			2	11
Fusarium acetilerea					1								1
Fusarium proliferatum								1					1
Fusarium solani	2												2
Fusarium sp.					1								1
Fusarium verticillioides							1						1
Geomyces sp.						1			1				2
Leptospora galii												1	1
Leptosphaeria sp.								1					1
Karstenula sp.												2	2
Naganishia diffluens												1	1
Magnaporthe oryzae				1									1
Massarina igniaria							1						1
Metarhizium anisopliae					1								1
Metschnikowia sp.									2				2
Microdochium bolleyi									2				2
Microsphaeropsis olivacea	3					1	1					1	6
Moesziomyces bullatus				1				1					2
Mucor circinelloides		5											5
Naganishia globosa	2												2
Naganishia uzbekistanensis												1	1
Neurospora sitophila							1						1
Neurospora tetrasperma					2								2
Nigrospora oryzae					3								3
Nigrospora sp.				1	1								2
Nothophoma spiraeae									1				1
Parapyrenochaeta sp.												1	1
Paramyrothecium tellicola									1				1
Penicillium commune										1			1
Penicillium concavorugulosum						2							2

Penicillium expansum							1				1		2
Penicillium goetzii		1						1					2
Penicillium granulatum						2							2
Penicillium nalgiovense						1							1
Penicillium oxalicum				2	1			2	1		3	1	10
Peniophora pseudoversicolor												1	1
Periconia sp.				3					2				4
Periconia lateralis											2		2
Penicillium sp.		1					1	1		3	1	1	8
Periconia igniaria								1					1
Periconia sahariana				1									1
Preussia similis												1	1
Periconia variicolor									1				1
Phaeobotryon negundinis							1						1
Phaeosphaeria typharum												1	1
Phanerochaete chrysosporium				2									2
Phoma macrostoma	3												3
Phoma medicaginis	1												1
Phoma medicaginis		1											1
Phoma sp.	1							1	1				3
Pithomyces sacchari				1									1
Pseudopithomyces chartarum											2		2
Pyrenochaetopsis confluens									1				1
Rhizopus sp.						1							1
Schizothecium inaequale		1											1
Schizophyllum commune												1	1
Scopulariopsis brevicaulis						1							1
Spegazzinia sp.							1						1
Sporormiella megalospora												1	1
Sporobolomyces carnicolor								1					1
Stachybotrys chartarum									2		1		3

Stagonosporopsis cucurbitacearum								1				1
Symmetrospora oryzicola								1				1
Symmetrospora symmetrica				1		1						2
Talaromyces amestolkiae							1					1
Talaromyces funiculosus						1	3	2		2	1	9
Talaromyces radicus					2					1		3
Talaromyces sp.					4		1	1				6
Talaromyces purpureogenus										1		1
Talaromyces verruculosus						1		1				2
Thielavia subthermophila											1	1
Torula caligans					2							2
Torula mackenziei						1		1		2		4
Torula sp.						1		2				3
Trichoderma sp.					1							1
Trichoderma longibrachiatum										1		1
Tricholoma matsutake	1											1
Trichurus spiralis								1				1
Ustilago levis					1							1
Vishniacozyma sp.						1						1

Table S2. Total number of fungal colonies isolated from each location from May 2020 to April 2021.

May-20																	
**Fungal species**		**1F**			**2F**				**3F**			**5F**			**6F**		**Total**
	**OD**	**Corridor**	**Washroom**	**OD**	**Corridor**	**Washroom**	**CA**	**OD**	**Corridor**	**Washroom**	**OD**	**Corridor**	**Washroom**	**OD**	**Corridor**	**Washroom**	
Alternaria alternata	6	0	5	6	1	3	0	4	1	5	7	0	2	4	2	0	46
Alternaria tenuissima	1	0	1	0	1	2	0	0	0	1	1	0	0	0	0	0	7
Alternaria sp.	0	1	1	0	0	0	0	0	0	0	0	0	0	1	0	0	3
Alternaria arborescens	0	0	1	0	0	0	0	0	0	0	0	0	0	0	0	0	1
Aspergillus niger	0	0		0	0	0	0	0	0	0	0	0	1	4	0	0	5
Alternaria doliconidium						1											1
Alternaria destruens		0		0	0	0	0	0	0	1	1	0	0	0	0	0	2
Aspergillus nomius								1					1				2
Microsphaeropsis olivacea	1	0		0	0	0	0	0	1	0	0	1	0	0	0	0	3
Phoma macrostoma	1			0	1	0	0	0	0	0	0	1	0	0	0	0	3
Cladosporium ramotenellum	0	1		0	0	0	0	0	0	0	0	1	0	0	0	0	2
Cladosporium cladosporioides				1													1
Phoma sp.										1							1
Didymella subherbarum										1							1
Phoma medicaginis										1							1
Naganishia globosa		1						1									2
Fusarium solani											1				1		2
Total	9	3	8	7	3	6	0	6	2	10	10	3	4	9	3	0	83

Jun-20																	
**Fungal species**		**1F**			**2F**				**3F**			**5F**			**6F**		
	OD	Corridor	Washroom	OD	Corridor	Washroom	CA	OD	Corridor	Washroom	OD	Corridor	Washroom	OD	Corridor	Washroom	Total
Alternaria alternata	1	1	1	3	1	1	1	2		4	2	3	1	7			28
Alternaria tenuissima									2								2
Alternaria pharbitidis							1										1
Alternaria sp.						1							1				2
Aspergillus niger												4					4

Aspergillus versicolor												1					1
Aureobasidium sp.														1			1
Phoma medicaginis								1									1
Cladosporium sp.													1				1
Penicillium goetzii									1								1
Penicillium sp.													1				1
Filobasidium magnum									1								1
Schizothecium inaequale											1						1
Tricholoma matsutake							1										1
Mucor circinelloides										5							5
Total	1	1	1	3	1	2	3	3	4	9	3	8	4	8	0	0	51
**Jul-20**																	
**Fungal species**	**1F**			**2F**			**3F**				**5F**			**6F**			
	**OD**	**Corridor**	**Washroom**	**OD**	**Corridor**	**Washroom**	**CA**	**OD**	**Corridor**	**Washroom**	**OD**	**Corridor**	**Washroom**	**OD**	**Corridor**	**Washroom**	**Total**
Alternaria alternata		2	2			2		2	2	1		1	1	3			16
Alternaria tenuissima	2	1	1		1	3		3		1	2			1	2		17
Alternaria tamaricis		1															1
Alternaria quercus				1													1
Alternaria sp.						1						1					2
Cladosporium sp.					1												1
Cladosporium ramotenellum							1										1
Cladosporium gossypiicola					1							1					2
Cladosporium delicatulum									1			1		1			3
Cladosporium anthropophilum			1											1			2
Cladosporium tenuissimum								1					2	1			4
Cladosporium oxysporum			1														1
Filobasidium magnum												1					1
Total	2	4	5	1	3	6	1	6	3	2	2	5	3	7	2	0	52
**Aug-20**																	
**Fungal species**	**1F**			**2F**			**3F**				**5F**			**6F**			

	OD	Corridor	Washroom	OD	Corridor	Washroom	CA	OD	Corridor	Washroom	OD	Corridor	Washroom	OD	Corridor	Washroom	Total
Alternaria alternata	1		1	4	2					1				4			13
Alternaria tenuissima	3							1		1		1		2	3		11
Aspergillus niger															1		1
Aspergillus tubingensis	1									1							2
Aspergillus awamor										1							1
Aureobasidium pullulans								1									1
Arthrinium saccharicola			1														1
Cladosporium cladosporioides								1							1		2
Cladosporium anthropophilum	1			1													2
Cladosporium tenuissimum								1									1
Coprinopsis atramentaria												1					1
Cercospora canescens														1			1
Curvularia verruculosa														1			1
Curvularia lunata						1				1							2
Curvularia sp.				1													2
Diaporthe sp.	1																1
Didymella macrostoma				1													1
Diatrype stigma							1										1
Exserohilum rostratum														1			1
Pithomyces sacchari								1									1
Penicillium oxalicum				1					1								2
Periconia sahariana													1				1
Periconia sp.				2										1			3
Epicoccum sp.				1													1
Magnaporthe oryzae	1																1
Moesziomyces bullatus						1											1
Phanerochaete chrysosporium	1							1									2
Nigrospora sp.								1	1								1
Total	9	0	2	11	2	2	1	7	2	5	0	2	1	10	5	0	59

September 2020																
**Fungal species**	**1F**			**2F**				**3F**			**5F**			**6F**		
	**OD**	**Corridor**	**Washroom**	**OD**	**Corridor**	**Washroom**	**CA**	**OD**	**Corridor**	**Washroom**	**OD**	**Corridor**	**Washroom**	**OD**	**Corridor**	**Washroom**	**Total**
Alternaria alternata		1		1								1		1	1		5
Alternaria tenuissima	4		5	4			1	4			2		2	2			24
Alternaria sp.			1	2				2									5
Aspergillus tubingensis														1			1
Alternaria infectoria														2			2
Aureobasidium pullulans	1								3								4
Aureobasidium melanogenum	1																1
Arthrinium saccharicola														1			1
Cladosporium cladosporioides	1		1	1	4	3	1	1	2	1	3			2			20
Cladosporium sp.					1	1											2
Cladosporium austroafricanum				1													1
Cladosporium anthropophilum				1													1
Cladosporium ramotenellum														1	1		2
Cladosporium halotolerans							1										1
Cladosporium tenuissimum								1									1
Cladosporium sphaerospermum										1							1
Coniothyrium sp.														1			1
Cercospora nicotianae	1						1										2
Epicoccum nigrum													1				1
Penicillium oxalicum							1										1
Curvularia intermedia				1													1
Epicoccum sp.								1									1
Metarhizium anisopliae	1																1
Nigrospora sp.				1													1
Nigrospora oryzae						1				1			1				3
Neurospora tetrasperma						1	1										2
Fusarium acetilerea			1														1
Filobasidium magnum														1			1

Fusarium sp.								1									1
Symmetrospora symmetrica				1													1
Total	9	1	8	13	5	6	6	10	5	3	5	1	4	12	2	0	90

							October 2020										
**Fungal species**	**1F**			**2F**				**3F**			**5F**			**6F**			
	**OD**	**Corridor**	**Washroom**	**OD**	**Corridor**	**Washroom**	**CA**	**OD**	**Corridor**	**Washroom**	**OD**	**Corridor**	**Washroom**	**OD**	**Corridor**	**Washroom**	
Alternaria alternata				3		1											4
Alternaria tenuissima							1				1	1		1	2		6
Alternaria sp.					1												1
Aspergillus sydowii	1																1
Aspergillus protuberus												1					1
Aspergillus pseudoglaucus	1																1
Aspergillus costaricensis		1															1
Cladosporium cladosporioides		1			1		1		1	1	3	1		1	2		12
Cladosporium gossypiicola						1					1						2
Cladosporium anthropophilum														1	2		3
Cladosporium tenuissimum		1															1
Colletotrichum gloeosporioides										1							1
Deniquelata quercina										1							1
Geomyces sp.															1		1
Penicillium granulatum	1			1													2
Penicillium concavorugulosum										2							2
Penicillium nalgiovense											1						1
Microsphaeropsis olivacea							1										1
Rhizopus sp.								1									1
Talaromyces sp.			1							2							3
Talaromyces radicus	1									1							2
Talaromyces variabilis										1							1
Torula caligans					1									1			2
Trichoderma sp.				1													1

Scopulariopsis brevicaulis						1											1
Ustilago levis											1						1
Total	4	3	1	5	3	3	3	1	1	9	7	3	0	4	7	0	54

November 2020

**Fungal species**	**1F**			**2F**				**3F**			**5F**			**6F**			
	**OD**	**Corridor**	**Washroom**	**OD**	**Corridor**	**Washroom**	**CA**	**OD**	**Corridor**	**Washroom**	**OD**	**Corridor**	**Washroom**	**OD**	**Corridor**	**Washroom**	**Total**
Alternaria alternata	1							1					1				3
Alternaria longipes	1																1
Alternaria tenuissima													1	1			2
Aspergillus ochraceus		1															1
Arthrinium sp.					1									1			2
Arthrinium saccharicola											1						1
Arthrinium piptatheri				1													1
Aureobasidium sp.				2													2
Aureobasidium namibiae				1													1
Cladosporium cladosporioides				1						1	2			3			7
Cladosporium sp.	2													1			3
Cladosporium tenuissimum											1		2				3
Coniothyrium pyrinum	1							2									3
Coprinopsis atramentaria												1					1
Fusarium verticillioides					1												1
Penicillium sp.							1										1
Penicillium expansum														1			1
Phaeobotryon negundinis								1									1
Microsphaeropsis olivacea				1													1
Neurospora sitophila															1		1
Torula sp.														1			1
Talaromyces funiculosus								1									1
Torula mackenziei											1						1
Talaromyces verruculosus								1									1

Spegazzinia sp.							1										1
Vishniacozyma sp.								1									1
Massarina igniaria														1			1
Symmetrospora symmetrica											1						1
Total	5	1	0	6	2	0	2	7	0	1	6	1	4	9	1	0	45

December 2020																	
**Fungal species**	**1F**			**2F**				**3F**			**5F**			**6F**			**0**
	**OD**	**Corridor**	**Washroom**	**OD**	**Corridor**	**Washroom**	**CA**	**OD**	**Corridor**	**Washroom**	**OD**	**Corridor**	**Washroom**	**OD**	**Corridor**	**Washroom**	**Total**
Alternaria alternata	3	1		2				3			2			1			12
Aspergillus niger				1													1
Aspergillus tubingensis				1	1												2
Aspergillus protuberus					1												1
Arthrinium sp.				1													1
Arthrinium marii								2									2
Arthrinium saccharicola											1						1
Arthrinium malaysianum															1		1
Arthrinium phaeospermum	1																1
Aureobasidium sp.								1									1
Aureobasidium proteae				1													1
Cladosporium cladosporioides				1				4							2		7
Cladosporium sp.	2			1	1									4	1		9
Cladosporium austroafricanum	1			1													2
Cladosporium tenuissimum				1													1
Cladosporium ramotenellum											1			3	1		5
Cryptococcus flavescens				1													1
Cercospora arctii-ambrosiae											1						1
Eurotium sp.	1																1
Fusarium proliferatum														1			1
Epicoccum nigrum				1													1
Leptosphaeria sp.				1													1

Penicillium sp.	1																1
Penicillium oxalicum			1				1										2
Filobasidium magnum				1										4			5
Penicillium goetzii											1						1
Periconia igniaria								1									1
Phoma sp.	1																1
Moesziomyces bullatus											1						1
Talaromyces funiculosus	2							1									3
Talaromyces sp.	1																1
Talaromyces amestolkiae	1																1
Sporobolomyces carnicolor				1													1
Total	14	1	1	15	3	0	1	12	0	0	7	0	0	13	5	0	72

January 2021																	
**Fungal species**	**1F**			**2F**				**3F**			**5F**			**6F**			**0**
	**OD**	**Corridor**	**Washroom**	**OD**	**Corridor**	**Washroom**	**CA**	**OD**	**Corridor**	**Washroom**	**OD**	**Corridor**	**Washroom**	**OD**	**Corridor**	**Washroom**	**Total**
Alternaria alternata				2							1						3
Alternaria sp.	1		1	2				1						1			6
Arthrinium sp.										1							1
Acrostalagmus sp.														1			1
Cladosporium cladosporioides				3				2			1						6
Cladosporium anthropophilum				1													1
Cladosporium tenuissimum											1						1
Cladosporium colombiae				1													1
Dothiora europaea								1									1
Phoma sp.		1															1
Geomyces sp.				1													1
Eurotium sp.	1																1
Penicillium oxalicum	1																1
Periconia sp.	1																1
Periconia variicolor									1								1

Cercospora nicotianae											1						1
Filobasidium magnum				1													1
Paramyrothecium tellicola				1													1
Nothophoma spiraeae											1						1
Microdochium bolleyi								2									2
Metschnikowia sp.	1							1									2
Talaromyces funiculosus	1							1									2
Torula sp.				2													2
Talaromyces verruculosus				1													1
Torula mackenziei	1																1
Talaromyces sp.									1								1
Symmetrospora oryzicola											1						1
Stachybotrys chartarum								1						1			2
Stagonosporopsis cucurbitacearum														1			1
Pyrenochaetopsis confluens												1					1
Trichurus spiralis															1		1
Total	7	1	1	15	0	0	0	9	2	1	6	1	0	4	1	0	48

February 2021																	
**Fungal species**		**1F**			**2F**				**3F**			**5F**			**6F**		**0**
	**OD**	**Corridor**	**Washroom**	**OD**	**Corridor**	**Washroom**	**CA**	**OD**	**Corridor**	**Washroom**	**OD**	**Corridor**	**Washroom**	**OD**	**Corridor**	**Washroom**	**Total**
Alternaria alternata										1							1
Alternaria tenuissima								1			1						2
Arthrinium sp.	1																1
Aspergillus niger			1	1							1			1			4
Aspergillus stellatus								1									1
Arthrinium sp.								1									1
Aspergillus sp.	2																2
Cladosporium cladosporioides														2			2
Cladosporium sp.								1					1				2
Penicillium commune												1					1

Penicillium sp.										1	1			1			3
Total	3	0	1	1	0	0	0	4	0	2	3	1	1	4	0	0	20

**March 2021**																	
**Fungal species**	**1F**			**2F**				**3F**			**5F**			**6F**			
	**OD**	**Corridor**	**Washroom**	**OD**	**Corridor**	**Washroom**	**CA**	**OD**	**Corridor**	**Washroom**	**OD**	**Corridor**	**Washroom**	**OD**	**Corridor**	**Washroom**	**Total**
Alternaria alternata		2		1				1									4
Alternaria sp.											1						1
Alternaria tenuissima	1				4					1							6
Aspergillus niger				1													1
Cladosporium cladosporioides											2			1			3
Cladosporium sp.	1								1								2
Cystobasidium sp.							2										2
Didymocyrtis brachylaenae														1			1
Epicoccum layuense								1									1
Penicillium sp.											1						1
Penicillium oxalicum				1									2				3
Periconia lateralis						1		1									2
Penicillium expansum					1												1
Pseudopithomyces chartarum															2		2
Talaromyces funiculosus	1			1													2
Talaromyces purpureogenus																	1
Talaromyces radicus		1															1
Trichoderma longibrachiatum				1													1
Torula mackenziei							1		1								2
Stachybotrys chartarum														1			1
Total	3	3	0	5	5	1	3	3	2	1	4	0	2	3	2	0	38

**April 2021**																	
**Fungal species**	**1F**			**2F**				**3F**			**5F**			**6F**			
	**OD**	**Corridor**	**Washroom**	**OD**	**Corridor**	**Washroom**	**CA**	**OD**	**Corridor**	**Washroom**	**OD**	**Corridor**	**Washroom**	**OD**	**Corridor**	**Washroom**	**Total**

Alternaria alternata	1								1		3	5
Aspergillus versicolor	1											1
Alternaria tenuissima			2				2				2	6
Cladosporium cladosporioides		1		1								2
Cladosporium ramotenellum	1			1					1	1		4
Cladosporium sinuosum	1											1
Cladosporium colombiae									1			1
Cladosporium austroafricanum									1			1
Naganishia diffluens				1								1
Coniothyrium aleuritis				1								1
Coprinopsis atramentaria		1										1
Thielavia subthermophila		1										1
Comoclathris sp.				1								1
Cladosporium sp.			3		3			1				7
Cladosporium tenuissimum									1			1
Didymella pedeiae	2											2
Dothiorella gregaria		1								1		2
Sporormiella megalospora											1	1
Filobasidium magnum	1										1	2
Eurotium sp.			1									1
Karstenula sp.										1	1	2
Penicillium oxalicum	1											1
Penicillium sp.						1						1
Preussia similis											1	1
Naganishia uzbekistanensis			1									1
Parapyrenochaeta sp.			1									1
Phaeosphaeria typharum		1										1
Microsphaeropsis olivacea									1			1
Talaromyces funiculosus											1	1
Leptospora galii							1					1
Schizophyllum commune							1					1

Peniophora pseudoversicolor	1																1
Total	9	0	0	0	5	8	0	5	3	1	4	1	6	3	10	0	55

F = Floor, OD = Outdoor, CA = Coffee area.

Table S3. Environmental factors recorded in each sampling site at the time of sampling.

Month	Sampling Sites	Temperature (°C)	Humidity (%)	Wind speed（m/s）	Air Quality Index
May 2020	First floor outdoor	30.6	44.3	5.40	64.00
	First floor corridor	26.3	54		
	First floor washroom	27.3	51.8		
	Second floor outdoor	32	40.2		
	Second floor corridor	26.8	50.6		
	Second floor washroom	28.2	45.1		
	Student coffee area	27.2	50.6		
	Third floor outdoor	28.6	45.3		
	Third floor corridor	27.2	48.9		
	Third floor washroom	29.1	43.2		
	Fifth floor outdoor	29.7	43.5		
	Fifth floor corridor	28.3	45.2		
	Fifth floor washroom	30.1	43.4		
	Sixth floor outdoor	31	40		
	Sixth floor corridor	33.8	38.9		
June 2020	First floor outdoor	26.3	65.7	4.90	57.00
	First floor corridor	25.7	66		
	First floor washroom	25	70		
	Second floor outdoor	26.5	64		
	Second floor corridor	26.8	63.8		
	Second floor washroom	26.9	64.8		
	Student coffee area	27	62.8		
	Third floor outdoor	26.8	67.2		

	Third floor corridor	27.7	61.7		
	Third floor washroom	27.4	65.4		
	Fifth floor outdoor	27.9	64.7		
	Fifth floor corridor	27.6	61.5		
	Fifth floor washroom	27.8	62.4		
	Sixth floor outdoor	30.9	52.9		
	Sixth floor corridor	30.8	54.3		
July 2020	First floor outdoor	25.78	64.39	4.60	57.00
	First floor corridor	25.19	64.68		
	First floor washroom	24.5	68.6		
	Second floor outdoor	25.97	62.72		
	Second floor corridor	26.26	62.52		
	Second floor washroom	26.36	63.5		
	Student coffee area	26.46	61.54		
	Third floor outdoor	26.26	65.86		
	Third floor corridor	27.15	60.47		
	Third floor washroom	26.85	64.09		
	Fifth floor outdoor	27.34	63.40		
	Fifth floor corridor	27.04	60.27		
	Fifth floor washroom	27.24	61.15		
	Sixth floor outdoor	30.28	51.84		
	Sixth floor corridor	30.18	53.21		
	First floor outdoor	23.7	60.2		
	First floor corridor	23.1	68.2		
	First floor washroom	22.3	73.4		
	Second floor outdoor	23.9	66.3		

August 2020	Second floor corridor	21.8	62.9	3.20	71.00
	Second floor washroom	23.7	65.9		
	Student coffee area	21.4	62.8		
	Third floor outdoor	23.4	66.5		
	Third floor corridor	23.2	64.9		
	Third floor washroom	23.3	65.8		
	Fifth floor outdoor	24.1	61.7		
	Fifth floor corridor	22.8	58.6		
	Fifth floor washroom	23.3	66.3		
	Sixth floor outdoor	24.0	57.5		
	Sixth floor corridor	24.5	54.4		
September 2020	First floor outdoor	18	57.2	5.00	67.00
	First floor corridor	18.3	61.3		
	First floor washroom	18.1	63.3		
	Second floor outdoor	18.1	59.2		
	Second floor corridor	19.3	55.6		
	Second floor washroom	18.3	60.7		
	Student coffee area	18.1	60.2		
	Third floor outdoor	18.4	59.5		
	Third floor corridor	18.5	58.6		
	Third floor washroom	18.3	58.8		
	Fifth floor outdoor	18.2	46.8		
	Fifth floor corridor	18.8	52.9		
	Fifth floor washroom	18.4	52.7		
	Sixth floor outdoor	19.9	42		
	Sixth floor corridor	20.4	47.1		

October 2020	First floor outdoor	14.742	54.74	3.60	72.00
	First floor corridor	14.742	48.944		
	First floor washroom	14.742	55.062		
	Second floor outdoor	14.742	49.266		
	Second floor corridor	18.284	52.486		
	Second floor washroom	23.436	64.722		
	Student coffee area	19.572	76.958		
	Third floor outdoor	16.03	46.368		
	Third floor corridor	25.046	51.52		
	Third floor washroom	22.148	39.928		
	Fifth floor outdoor	16.03	42.182		
	Fifth floor Corridor	21.182	66.01		
	Fifth floor Washroom	26.334	65.366		
	Sixth floor outdoor	29.2	45.402		
	Sixth floor corridor	21.826	33.166		
November 2021	First floor outdoor	4.1	36.5	3.20	29.00
	First floor corridor	4.1	37		
	First floor washroom	4.1	37.1		
	Second floor outdoor	4.1	37.1		
	Second floor corridor	8.1	37.5		
	Second floor washroom	14.6	22.6		
	Student coffee area	14.2	26.6		
	Third floor outdoor	6.8	32.8		
	Third floor corridor	12.6	29.5		
	Third floor washroom	16.3	26.1		
	Fifth floor outdoor	6.3	34.8		

	Fifth floor corridor	13.8	24.4		
	Fifth floor washroom	16.2	24.3		
	Sixth floor outdoor	7.1	31.7		
	Sixth floor corridor	10.3	36.5		
	First floor outdoor	1.1	25.8		
	First floor corridor	3	25		
	First floor washroom	4.6	14.8		
	Second floor outdoor	1.4	24.7		
	Second floor corridor	11.4	14.6		
	Second floor washroom	14.3	20.6		
	Student coffee area	13.1	12.9		
December 2021	Third floor outdoor	3.7	18.8	3.50	63.00
	Third floor corridor	14.8	11.7		
	Third floor washroom	16.2	11.1		
	Fifth floor outdoor	3.8	21.9		
	Fifth floor corridor	15.1	25.1		
	Fifth floor washroom	14.9	26		
	Sixth floor outdoor	1.6	24.2		
	Sixth floor corridor	7.8	16.9		
	First floor outdoor	2.6	23.7		
	First floor Corridor	8.4	21.9		
	First floor washroom	9.5	23.1		
	Second floor outdoor	2.9	24.2		
January 2021	Second floor corridor	13	16	1.30	65.00
	Second floor washroom	11.7	20.9		
	Student coffee area	15.4	17.9		

	Third floor outdoor	3.8	22.9		
	Third floor corridor	15.3	17		
	Third floor washroom	16	16		
	Fifth floor outdoor	3.2	23.4		
	Fifth floor corridor	14.2	15.3		
	Fifth floor washroom	16	17.2		
	Sixth floor outdoor	11.0	18		
	Sixth floor corridor	5.3	20		
February 2021	First floor outdoor	6.1	82	1.50	47.00
	First floor corridor	10.3	45.9		
	First floor washroom	10.6	44		
	Second floor outdoor	5.7	67		
	Second floor corridor	9.9	48		
	Second floor washroom	10.1	50		
	Student coffee area	9.9	45.2		
	Third floor outdoor	6.7	59		
	Third floor corridor	11.3	36.2		
	Third floor washroom	10.7	46		
	Fifth floor outdoor	5.8	77.4		
	Fifth floor corridor	10.4	43.7		
	Fifth floor washroom	10.5	40		
	Sixth floor outdoor	5.6	77.3		
	Sixth floor corridor	10.6	41.5		
	First floor outdoor	8.6	41.8		
	First floor corridor	9.8	38.9		
	First floor washroom	10.5	41.7		

March 2021	Second floor outdoor	8.9	40.2	3.40	90.00
	Second floor corridor	10.7	33.9		
	Second floor washroom	10.7	34.7		
	Student coffee area	10.3	36.2		
	Third floor outdoor	9.9	36.2		
	Third floor corridor	11.9	30.4		
	Third floor washroom	11.9	33.6		
	Fifth floor outdoor	10.3	35.5		
	Fifth floor corridor	11.3	31.8		
	Fifth floor washroom	11.5	32.4		
	Sixth floor outdoor	10	32.6		
	Sixth floor corridor	11.6	32		
April 2021	First floor outdoor	15.1	70.2	3.50	70.00
	First floor corridor	16.4	65.6		
	First floor washroom	17.7	64.2		
	Second floor outdoor	16.1	66.6		
	Second floor corridor	19	56.6		
	Second floor washroom	17.6	61.6		
	Student coffee area	19.5	53.9		
	Third floor outdoor	15.5	68.2		
	Third floor corridor	18.1	60.2		
	Third floor washroom	18.7	58.6		
	Fifth floor outdoor	12.5	70.8		
	Fifth floor corridor	14.1	57.4		
	Fifth floor washroom	21.2	50.3		
	Sixth floor outdoor	12.4	73		

	Sixth floor corridor	14.6	54.6		

Table S4. Correlation analysis between fungal abundance and environmental parameters.

Fungal Genera	Factor	r	*p*-value
Alternaria	Relative humidity	0.226959253	0.0021851
Arthrinium	Relative humidity	-0.166102792	0.025848443
Aspergillus	Relative humidity	0.123018591	0.099923553
Aureobasidium	Relative humidity	-0.021899049	0.770443599
Cladosporium	Relative humidity	0.010135138	0.892585546
Filobasidium	Relative humidity	-0.020328471	0.786496421
Penicillium	Relative humidity	-0.050647368	0.499545325
Phoma	Relative humidity	-0.053211422	0.478054348
Talaromyces	Relative humidity	-0.218006116	0.003282896
Torula	Relative humidity	-0.167355753	0.024734031
Alternaria	Temperature	0.417569296	5.48037E-09
Arthrinium	Temperature	-0.313949484	1.77274E-05
Aspergillus	Temperature	-0.072864399	0.331020314
Aureobasidium	Temperature	-0.063003142	0.400783826
Cladosporium	Temperature	-0.092288379	0.217880398
Filobasidium	Temperature	-0.074759049	0.318567096
Penicillium	Temperature	-0.205390172	0.005675683
Phoma	Temperature	0.113599094	0.128915279
Talaromyces	Temperature	-0.29789416	4.87746E-05
Torula	Temperature	-0.16035195	0.031532016

Table S5. Relative abundances of top ten genera in five sampling floors.

Floor	Phylum	Class	Genus	Relative abundance (%)
1F	Ascomycota	Dothideomycetes	*Alternaria*	54.36893204
1F	Ascomycota	Dothideomycetes	*Cladosporium*	15.53398058
1F	Ascomycota	Eurotiomycetes	*Aspergillus*	8.737864078
1F	Ascomycota	Eurotiomycetes	*Penicillium*	4.854368932
1F	Ascomycota	Eurotiomycetes	*Talaromyces*	8.737864078
1F	Ascomycota	Sordariomycetes	*Arthrinium*	2.912621359
1F	Ascomycota	Dothideomycetes	*Aureobasidium*	1.941747573
1F	Basidiomycota	Tremellomycetes	*Filobasidium*	0.970873786
1F	Ascomycota	Dothideomycetes	*Periconia*	0.970873786
1F	Ascomycota	Dothideomycetes	*Torula*	0.970873786
2F	Ascomycota	Dothideomycetes	*Alternaria*	48.09160305
2F	Ascomycota	Dothideomycetes	*Cladosporium*	28.24427481
2F	Ascomycota	Eurotiomycetes	*Aspergillus*	4.580152672
2F	Ascomycota	Eurotiomycetes	*Penicillium*	5.34351145
2F	Ascomycota	Eurotiomycetes	*Talaromyces*	1.526717557
2F	Ascomycota	Sordariomycetes	*Arthrinium*	2.290076336
2F	Ascomycota	Dothideomycetes	*Aureobasidium*	3.053435115
2F	Basidiomycota	Tremellomycetes	*Filobasidium*	1.526717557
2F	Ascomycota	Dothideomycetes	*Periconia*	2.290076336
2F	Ascomycota	Dothideomycetes	*Torula*	3.053435115
3F	Ascomycota	Dothideomycetes	*Alternaria*	44.33962264
3F	Ascomycota	Dothideomycetes	*Cladosporium*	24.52830189
3F	Ascomycota	Eurotiomycetes	*Aspergillus*	3.773584906
3F	Ascomycota	Eurotiomycetes	*Penicillium*	5.660377358

Floor	Phylum	Class	Genus	Relative abundance (%)
3F	Ascomycota	Eurotiomycetes	*Talaromyces*	8.490566038
3F	Ascomycota	Sordariomycetes	*Arthrinium*	3.773584906
3F	Ascomycota	Dothideomycetes	*Aureobasidium*	4.716981132
3F	Basidiomycota	Tremellomycetes	*Filobasidium*	0.943396226
3F	Ascomycota	Dothideomycetes	*Periconia*	2.830188679
3F	Ascomycota	Dothideomycetes	*Torula*	0.943396226
5F	Ascomycota	Dothideomycetes	*Alternaria*	43.61702128
5F	Ascomycota	Dothideomycetes	*Cladosporium*	31.91489362
5F	Ascomycota	Eurotiomycetes	*Aspergillus*	9.574468085
5F	Ascomycota	Eurotiomycetes	*Penicillium*	8.510638298
5F	Ascomycota	Eurotiomycetes	*Talaromyces*	1.063829787
5F	Ascomycota	Sordariomycetes	*Arthrinium*	2.127659574
5F	Ascomycota	Dothideomycetes	*Aureobasidium*	0
5F	Basidiomycota	Tremellomycetes	*Filobasidium*	1.063829787
5F	Ascomycota	Dothideomycetes	*Periconia*	1.063829787
5F	Ascomycota	Dothideomycetes	*Torula*	1.063829787
6F	Ascomycota	Dothideomycetes	*Alternaria*	45.09803922
6F	Ascomycota	Dothideomycetes	*Cladosporium*	32.35294118
6F	Ascomycota	Eurotiomycetes	*Aspergillus*	6.862745098
6F	Ascomycota	Eurotiomycetes	*Penicillium*	1.960784314
6F	Ascomycota	Eurotiomycetes	*Talaromyces*	0.980392157
6F	Ascomycota	Sordariomycetes	*Arthrinium*	2.941176471
6F	Ascomycota	Dothideomycetes	*Aureobasidium*	0.980392157
6F	Basidiomycota	Tremellomycetes	*Filobasidium*	5.882352941
6F	Ascomycota	Dothideomycetes	*Periconia*	0.980392157

6F	Ascomycota	Dothideomycetes	*Torula*	1.960784314
